# Neural crest E-cadherin loss drives cleft lip/palate by epigenetic modulation via pro-inflammatory gene–environment interaction

**DOI:** 10.1038/s41467-023-38526-1

**Published:** 2023-05-24

**Authors:** Lucas Alvizi, Diogo Nani, Luciano Abreu Brito, Gerson Shigeru Kobayashi, Maria Rita Passos-Bueno, Roberto Mayor

**Affiliations:** 1 Department of Cell and Developmental Biology, University College London, Gower Street, London, WC1E 6BT UK; 2 Centro de Estudos do Genoma Humano e Celulas-Tronco, Departamento de Genetica e Biologia Evolutiva, Instituto de Biociencias, Universidade de Sao Paulo, Sao Paulo, Brazil; 3 Center for Integrative Biology, Faculty of Sciences, Universidad Mayor, Santiago, Chile

**Keywords:** Disease model, DNA methylation, Cleft lip and palate, Cadherins

## Abstract

Gene–environment interactions are believed to play a role in multifactorial phenotypes, although poorly described mechanistically. Cleft lip/palate (CLP), the most common craniofacial malformation, has been associated with both genetic and environmental factors, with little gene–environment interaction experimentally demonstrated. Here, we study CLP families harbouring *CDH1*/E-Cadherin variants with incomplete penetrance and we explore the association of pro-inflammatory conditions to CLP. By studying neural crest (NC) from mouse, *Xenopus* and humans, we show that CLP can be explained by a 2-hit model, where NC migration is impaired by a combination of genetic (*CDH1* loss-of-function) and environmental (pro-inflammatory activation) factors, leading to CLP. Finally, using in vivo targeted methylation assays, we demonstrate that *CDH1* hypermethylation is the major target of the pro-inflammatory response, and a direct regulator of E-cadherin levels and NC migration. These results unveil a gene–environment interaction during craniofacial development and provide a 2-hit mechanism to explain cleft lip/palate aetiology.

## Abstract (web summary)

Cleft lip and palate is a common birth defect thought to involve both genetic and environmental components in its etiology. Here they identify a mechanism involving inflammation and E-cadherin mutations that reduces neural crest migration, leading to craniofacial defects.

## Introduction

The concept of gene–environment interactions has been present in genetics for decades^1–6^, however with scarce demonstration of how this interaction occurs in human traits, especially in regards to development. Gene–environment interactions studies have mostly focused on environmental insults during development leading to maladaptive development and metabolic disorders^2–4^, with rare contributions to human malformation.

Cleft lip with or without cleft palate (CLP), the most frequent craniofacial malformation in humans (1:700 live-births) has been widely described as a multifactorial condition affecting craniofacial development^7–9^. Although CLP heritability studies corroborate the role of both genetic and environmental factors in its aetiology, with heritability ranging from 45 to 85% depending on the analysed population^10,11^, most studies rely either on associating genetic variants to CLP by sequencing strategies or on epidemiological association of environmental variables to CLP^8,9,12,13^.

Candidate genes associated with CLP tend to fall into the Epithelial-to-Mesenchymal transition (EMT) pathway and pathogenic variants in genes belonging to the cadherin-catenin complex have been linked to familial CLP^14–18^. *CDH1* (E-cadherin), a member of the cadherin-catenin complex, is an example in which pathogenic variants can lead to CLP and/or gastric cancer. Despite the progress made on the identification of predisposing genetic variation to CLP, which includes both rare pathogenic variants as well as common single nucleotide polymorphisms (SNPs), those variants fail to completely explain CLP heritability, as in many complex disorders^19–21^. Alongside this missing heritability scenario, a similar phenomenon is also displayed in familial CLP harbouring rare pathogenic variants, in which incomplete penetrance is often observed and variants do not fully explain the phenotype, which remains an old question still not fully answered^22–28^. Incomplete penetrance is also the case for *CDH1*-linked CLP^17^, suggesting additional factors may play a role in this malformation.

On the other hand, epidemiological studies have associated pro-inflammatory conditions as diabetes, maternal infections, and obesity to CLP^29–37^, however, with no experimental demonstration of how those risk factor could affect craniofacial development. Concurrently, there is evidence that pro-inflammatory activation modulates *CDH1*/E-cadherin levels in multiple cell models^38–45^.

Therefore, we have explored the gene–environment interface leading to impairments in craniofacial development taking into account four key observations: (1) CLP families harbouring *CDH1*/E-cadherin pathogenic variants exhibit incomplete penetrance; (2) epidemiological associations linking maternal pro-inflammatory conditions to a higher risk of CLP in the offspring^12–20^; (3) cellular and molecular evidence of *CDH1*/E-cadherin downregulation under pro-inflammatory cytokine or bacteria exposure^21–26^; and (4) *CDH1*-linked familial gastric cancer, in which *CDH1* promoter hypermethylation acts as a second hit for penetrance^27,28^. We therefore hypothesised a 2-hit model in which CLP penetrance is dependent on a genetic hit (*CDH1* loss-of-function) and an environmental hit (pro-inflammatory activation) leading to *CDH1* epigenetic variation. In this study, we investigated craniofacial development-associated phenotypes resulting from the combination of gene loss-of-function with environmental insults using both in vitro and in vivo models.

## Results

### Neural crest E-cadherin loss leads to clefting phenotype

**Fig. 1:**
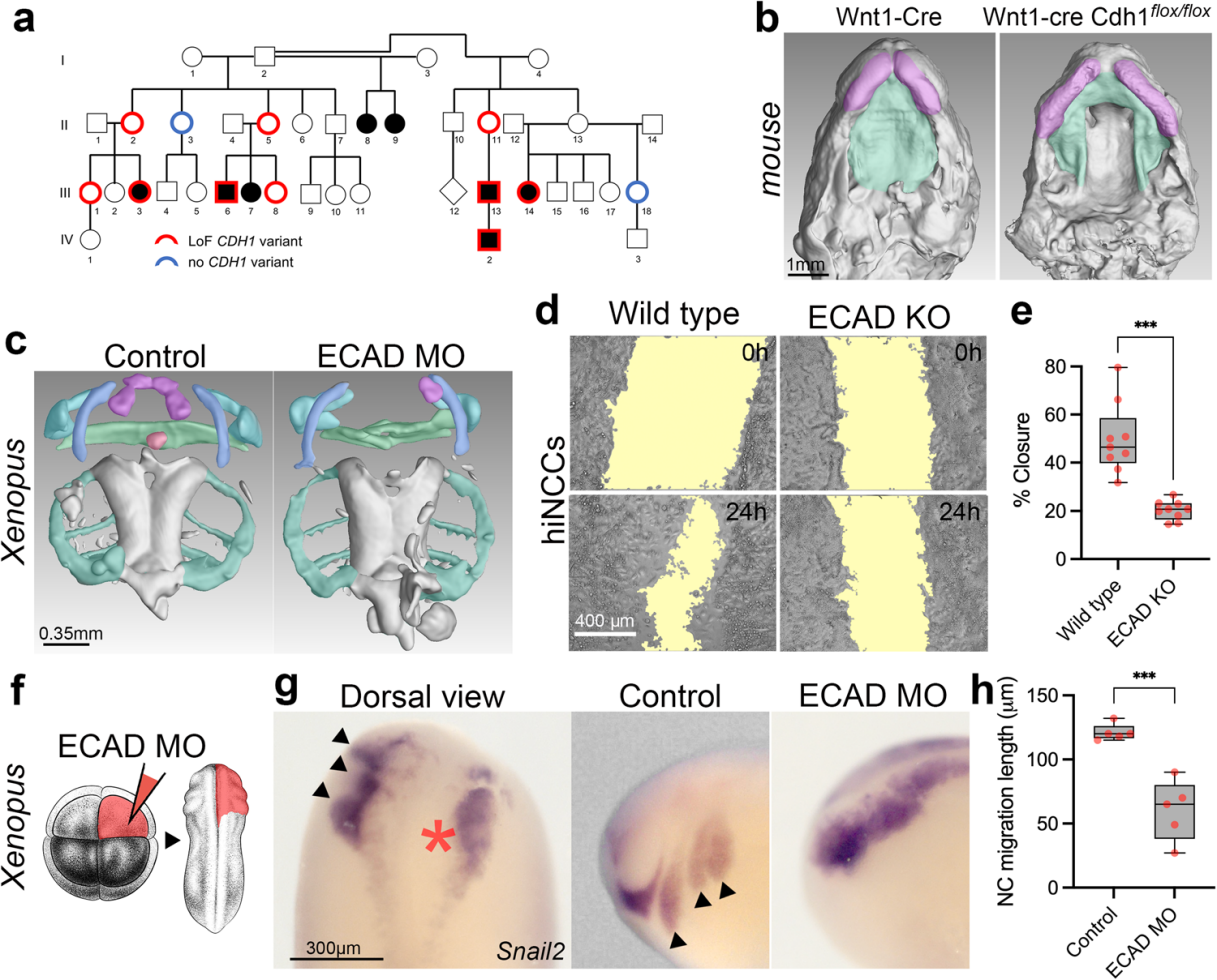
*CDH1*-linked oral clefting is consistent with E-cadherin loss in the neural crest. **a** Example of *CDH1*-linked CLP pedigree (Family F3788) displaying a loss-of-function (LoF) *CDH1* heterozygous variant c.760G>A (p.(Asp254Asn)) and CLP. In red, individuals with the LoF *CDH1* variant and without (blue). Non-penetrant individuals (II-2, II-5, II-11, III-1, III-8). Individuals with no red or blue borders were not tested^17^. **b** Transversal sections in μCT 3-dimensional reconstructions from Wnt1-Cre2 (*n* = 3) and Wnt1-Cre2 *Cdh1*^flox/flox^ (*n* = 5) E15.5 mice showing palatal shelves (green) and upper lip (magenta). **c** μCT 3D reconstructions of craniofacial cartilages from controls (*n* = 12) and E-cadherin morpholino knockdown (ECAD MO, *n* = 15) *Xenopus laevis* larvae at stage 52. Infrarostral cartilage (magenta) are mostly absent in ECAD MO and superostral cartilage (green) is malformed. 14 out of 15 (93%) of larvae displayed such observed phenotypes. **d** Scratch assays in wild type and ECAD KO hiNCCs evidencing a larger gap in ECAD KO hiNCCs after 24 h (scratch area in yellow) and **e** quantification of percentage of scratch closure after 24 h in wild type (*n* = 9) and ECAD KO (*n* = 9) hiNCCs, with less closure in ECAD KO (*p* = 0.0002, **f** Illustration of ECAD MO injections in 8-cell stage embryos targeting the neural crest at the right side of the embryo (in red). *Xenopus* illustrations © Natalya Zahn (2022). **g** *snai2* RNA in situ hybridisations evidencing *Xenopus* neural crest migration in non-injected side and ECAD MO injected side. In the dorsal view, neural crest migrates normally at the non-injected side (black arrows) and presents impaired migration at the ECAD MO injected side (red asterisk). Lateral views are also displayed. **h** Quantification of neural crest (NC) migration lengths in controls (*n* = 5) and ECAD MO injected embryos (*n* = 5) with impaired migration in ECAD MO (*p* = 0.00026, two-sided Welch’s *t* test). Boxplots centre is the median, with bounds representing the 25th and 75th percentile, and whiskers as minima to maxima. Source data are provided as a Source Data file.

Based on the observation that families harbouring *CDH1*/E-cadherin pathogenic variants show CLP with incomplete penetrance (~53%) (Fig. 1a, Supplementary Fig. 1, Supplementary Table1) we decided to explore the role of *CDH1*/E-cadherin on CLP ethology. Although most of craniofacial development is dependent on neural crest^46–48^ a requirement for E-cadherin during cranial neural crest migration has remained controversial^49,50^. We asked whether a lack of E-cadherin in the neural crest is compatible with CLP phenotypes. Using mice expressing Cre recombinase under the neural crest-specific *Wnt1* promoter (*Wnt1-Cre2*) and floxed *Cdh1*-LoxP, we found cleft palate (100% penetrance, *n* = 5) and wider lip gaps (80% penetrance, 4 out of *n* = 5) in *Wnt1-Cre2*/*Cdh1*^flox/flox^ E15.5 mouse embryos (Fig. 1b; Supplementary Fig. 2). Double heterozygous (*Wnt1-Cre2/Cdh1*^flox/+^) or floxed homozygous lacking *Wnt1-Cre2* (*Cdh1*^flox/flox^) did not shown any observable craniofacial phenotype. Such findings show that oral clefting phenotypes linked to *CDH1* loss-of-function could be neural crest related. To study neural crest development in vivo we used a previously characterised E-cadherin morpholino knockdown in *Xenopus laevis*^50^ by injecting blastomeres which give rise mainly to neural crest. Neural crest depleted of E-cadherin also exhibited craniofacial phenotypes affecting mainly upper and lower jaw cartilages in 93% of morphant *Xenopus* larvae (Fig. 1c). These phenotypes in neural crest derivatives could be due to problems in neural crest formation, migration, or differentiation. In order to test whether such phenotypes were neural crest migration related, we performed E-cadherin morpholino knockdown in the neural crest in *Xenopus* and observed significant reduction in in vivo migration (Fig. 1f–h). Additionally, E-cadherin morpholino co-injected with human wild type E-cadherin mRNA was able to rescue neural crest migration, whereas co-injection with E-cadherin mRNAs bearing the familial CLP variants here reported fail to rescue neural crest migration in vivo (Supplementary Fig. 3). To test whether these observations in animal models can be reproduced in human neural crest cells, we performed in vitro migration assays using human induced neural crest cells (hiNCCs) from iPSCs with CRISPR-Cas9 edited for *CDH1* loss-of-function. Using scratch assays in hiNCCs and *CDH1* loss-of-function (ECAD KO/21del ECAD), we observed that hiNCCs migration was significantly impaired by *CDH1* loss (Fig. 1d–e), consistent with the reduced migration observed in *Xenopus* neural crest (Fig. 1f–h). Therefore both in vivo (*Xenopus* embryos) and in vitro (human cells) manipulations of *CDH1*/E-cadherin in the neural crest presented significant neural crest migration reduction (Fig. 1d–h). Taken together, these results demonstrate that E-cadherin loss-of-function affects neural crest migration and that such neural crest E-cadherin loss is compatible with craniofacial phenotypes such as CLP.

### Pro-inflammatory activation impairs neural crest migration

**Fig. 2:**
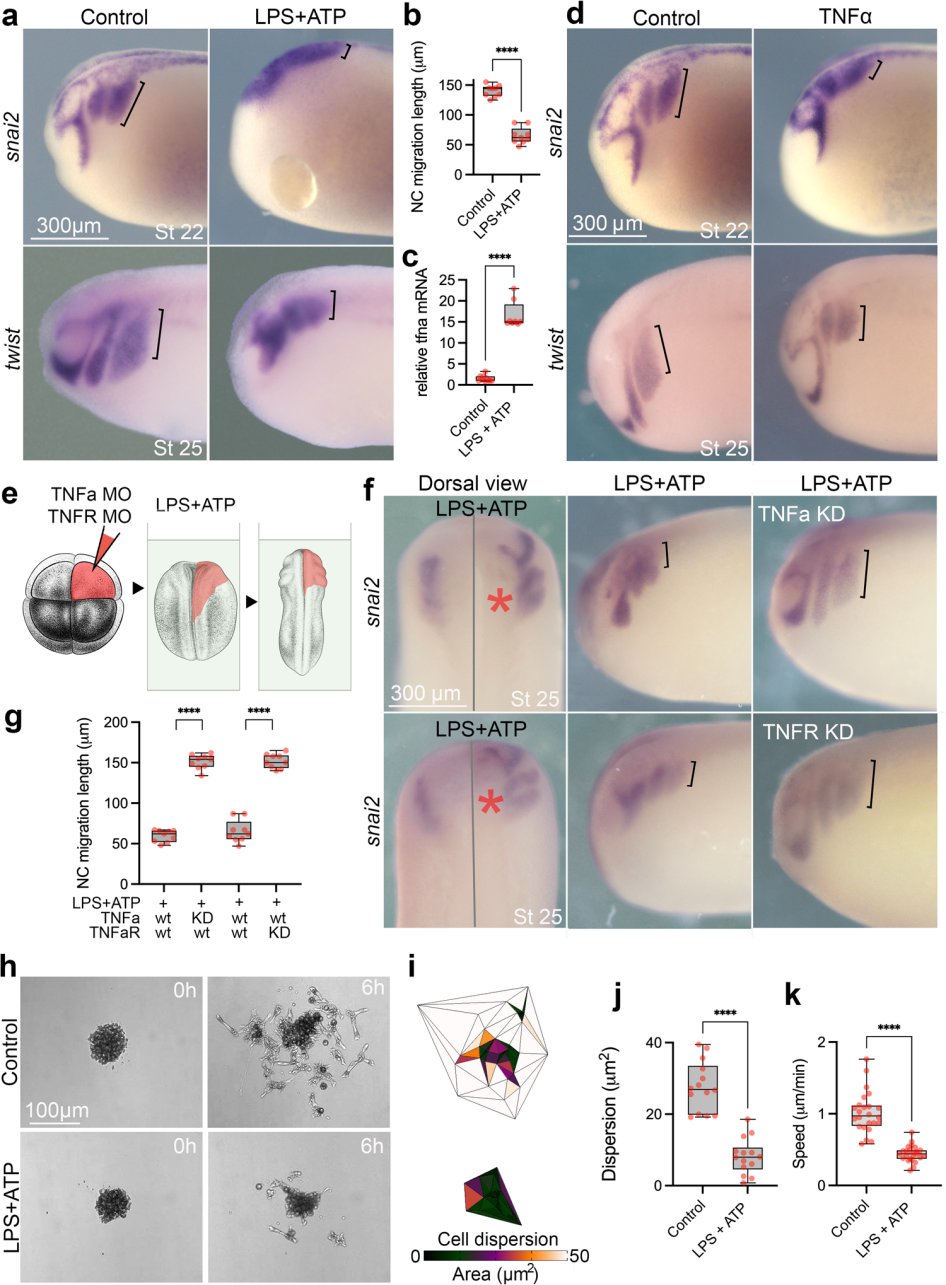
Pro-inflammatory activation inhibits neural crest migration in vivo and in vitro. **a** RNA in situ hybridisations (ISH) evidencing neural crest migration for *snai2* and *twist* in controls and LPS + ATP treated embryos. Neural crest migration is evidenced by black brackets. **b** Neural crest migration quantification in *snai2* ISH shows significant reduction in LPS + ATP treated embryos (*n* = 9) in comparison to controls (*n* = 9) (*p* = 0.0003, two-sided Welch’s *t* test). **c** *tnfa* expression in controls (*n* = 10) and LPS + ATP (*n* = 8) neural crest explants, with significant upregulation in LPS + ATP treated embryos (*p* = 0.0001, two-sided Welch’s *t* test). **d** *snai2* and *twist* ISH of controls and TNFa protein injected embryos displaying reduced neural crest migration. **e** Illustration of TNFa morpholino (TNFa MO) and TNFR morpholino (TNFR MO) injections at 8-cell stage targeting the right-side neural crest (in red) followed by LPS + ATP treatment. *Xenopus* illustrations © Natalya Zahn (2022). **f** Dorsal and lateral views from *snai2* ISH in TNFa MO and TNFR MO injected embryos upon LPS + ATP treatment. *Injected side. **g** Neural crest migration quantification in LPS + ATP exposed embryos with or without TNF MO or TNFR MO knockdowns (KD) showing significant rescue of migration in TNF MO and TNFR MO injected sides (*n* = 9; *p* < 0.0001, Two-way ANOVA). **h** in vitro neural crest migration of *Xenopus* neural crest explants under control (*n* = 14) or LPS + ATP (*n* = 14) treatment displaying reduced cell dispersion in LPS + ATP. **i** Triangulation analysis of neural crest in vitro dispersion showing reduced areas in LPS + ATP treated embryos. **j** Cell dispersion is reduced in LPS + ATP treated explants (*n* = 14) in comparison to controls (*n* = 14) (*p* < 0.0001, two-sided Welch’s *t* test). **k** Neural crest cells under LPS + ATP treatment with reduced speed of migration (LPS + ATP *n* = 24; control *n* = 24; *p* < 0.0001, two-sided Welch’s *t* test). Boxplots centre is the median, with bounds representing the 25th and 75th percentile, with and whiskers as minima to maxima. Source data are provided as a Source Data file. *p < 0.05, **p < 0.01, ***p < 0.001 and ****p < 0.0001.

We next evaluated whether pro-inflammatory activation could affect in vivo neural crest migration. Importantly, neural crest cells have been reported to respond to and produce pro-inflammatory cytokines both in vitro and in vivo^37,51–53^. Exposing pre-migratory neural crest *Xenopus* embryos (stage 15) to a well-characterised pro-inflammatory stimulus combination of lipopolysaccharide (LPS) and adenosine triphosphate (ATP)^54,55^) impairs neural crest migration (Fig. 2a, b; Supplementary Fig. 4a). We tested the pro-inflammatory response by showing an upregulation of *tnfa* mRNA in the neural crest of LPS + ATP treated embryos, as well as pro-inflammatory cytokines *il1b, il6* and NFkB component *nfkb1* (Fig. 2c, Supplementary Fig. 5a). Similar reduction in neural migration is also observed when TNFa protein is injected into the neural crest region of *Xenopus* embryos (Fig. 2d; Supplementary Fig. 4b, c). To test the role of the TNFa pathway in neural crest migration, *Xenopus* embryos depleted of TNFa or TNFR were treated with LPS and ATP (Fig. 2e). The inhibition of neural crest migration induced by LPS/ATP treatment was reversed by TNFa/TNFR inhibition (Fig. 2f, g), showing that the proinflammatory response is TNFa-dependent. In our in vivo pro-inflammatory model, we also observed that LPS + ATP is more efficient in upregulating *tnfa* mRNA than LPS or ATP alone (Supplementary Fig. 5b). To test if the in vivo migratory phenotypes in the neural crest were cell autonomous, we directly treated isolated neural crest with LPS + ATP. While control neural crest explants dispersed as expected, LPS + ATP treated cells show reduced dispersion and speed of migration (Fig. 2h–k), indicating that neural crest cells can directly respond to the proinflammatory stimulus. Both in vivo and in vitro phenotypes led us to conclude that the neural crest can respond to external pro-inflammatory stimuli by cytokine upregulation and changes in migratory behaviour.

### Neural crest E-cadherin is downregulated by pro-inflammation

**Fig. 3:**
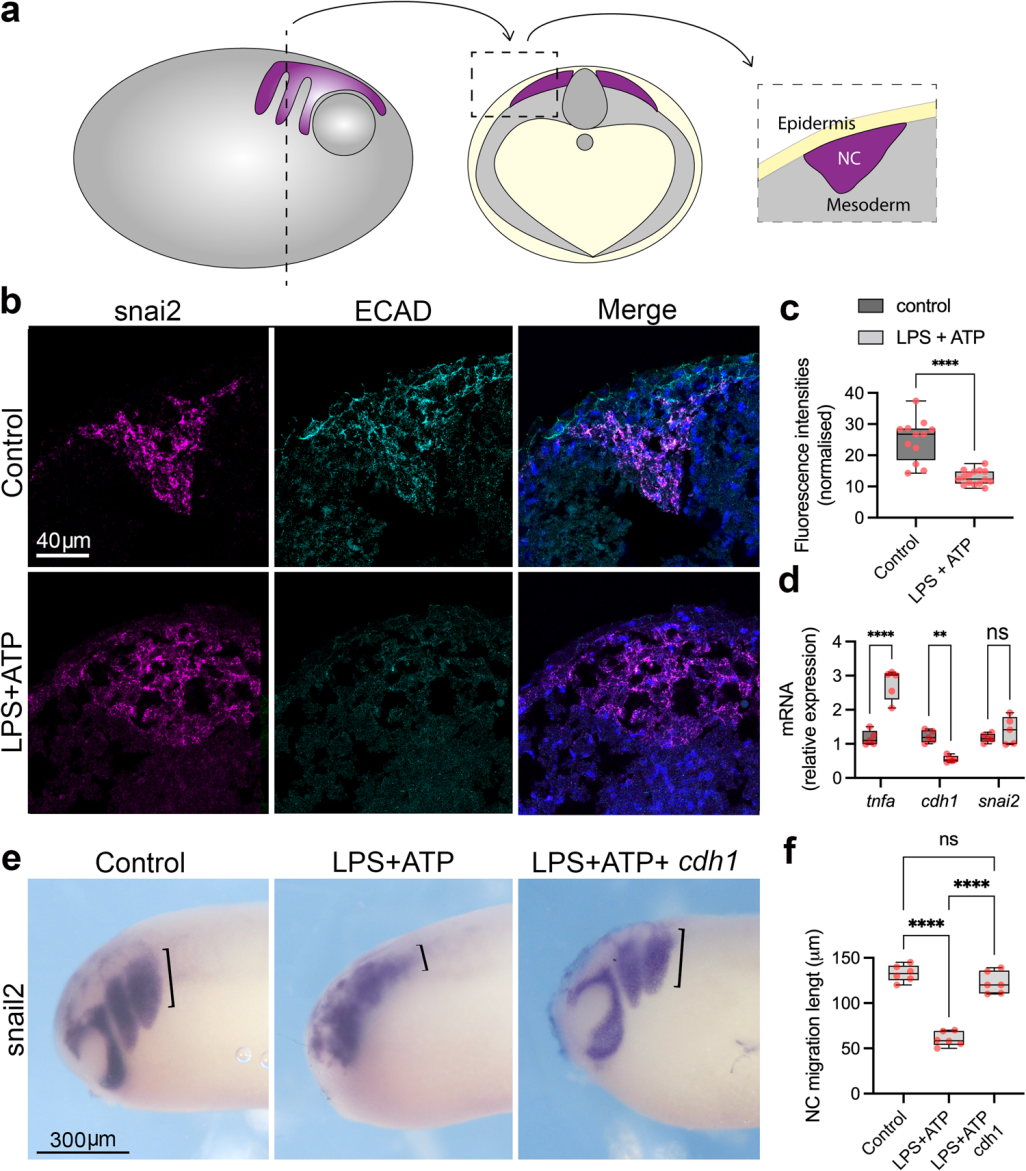
Pro-inflammatory activation leads to E-cadherin downregulation in the neural crest. **a** Scheme of cryosections for immunofluorescence in the neural crest, in which embryos were sectioned about 300um from the anterior end. Neural crest is represented in purple, mesoderm in grey and epidermis in yellow. **b** *snai2* fluorescent ISH (magenta) combined with E-cadherin (ECAD) immunofluorescence (cyan) depicting ECAD levels in the neural crest in control and LPS + ATP conditions. Merged images with DAPI (blue). E-cadherin signal is reduced on the *snai2* positive area in LPS + ATP treated embryos. **c** Quantification of E-cadherin fluorescence shown in b (Control *n* = 12, LPS + ATP *n* = 15, *p* < 0.0001). **d** qPCR quantification of *tnfa*, *cdh1* and *snai2* expression levels in neural crest explants from control (*n* = 5) and LPS + ATP (*n* = 5) treated embryos, showing significant upregulation of tnfa (*p* < 0.0001), significant downregulation of cdh1 (*p* = 0.0031) and non-significant changes in snai2 (*p* = 0.5290) (Two-way ANOVA). **e** snai2 ISH evidencing neural crest migration in control, LPS + ATP and LPS + ATP+*cdh1* mRNA. *cdh1* mRNA injections can partially rescue impaired neural crest migration caused by LPS + ATP pro-inflammatory activation. Neural crest migration lengths are evidenced by black brackets. **f** Neural crest migratory phenotype rescue in LPS + ATP (*N* = 6) and LPS + ATP+*cdh1* mRNA (*N* = 6) embryos, with significant higher rescue frequencies in LPS + ATP+*cdh1* mRNA (*p* < 0.0001, one-way ANOVA). *p < 0.05, **p < 0.01, ***p < 0.001 and ****p < 0.0001. Boxplots centre is the median, with bounds representing the 25th and 75th percentile, with and whiskers as minima to maxima. Source data are provided as a Source Data file.

Pro-inflammatory activation either by bacterial infection or direct pro-inflammatory cytokine exposure can lead to E-cadherin downregulation in a variety of cellular models^38–43^. Therefore, we evaluated *cdh1*/E-cadherin expression in the *Xenopus* migrating neural crest and observed a reduction in both protein E-cadherin and *cdh1* mRNA levels upon the pro-inflammatory treatment (Fig. 3a–d), with no effect on the expression of the neural crest marker *snai2* and upregulation of the pro-inflammatory factor *tnfa, as expected* (Fig. 3d). Because E-cadherin knockdown in the *Xenopus* neural crest led to reduced migration and LPS + ATP treatment resulted in E-cadherin downregulation, we tested whether pro-inflammatory phenotypes could be E-cadherin related. We therefore overexpressed *cdh1* by *cdh1* mRNA injection in the neural crest precursor blastomeres and exposed embryos to LPS + ATP, followed by neural crest migration analysis. *cdh1* mRNA overexpression was able to rescue the neural crest migratory phenotype in comparison to non-injected embryos (Fig. 3e, f). This result indicates that E-cadherin expression is the major downstream target of pro-inflammatory activation with respect to neural crest migration; however, we cannot rule out that other genes not analysed in this study could also play a role in the pro-inflammatory phenotype, as other cadherins are known to be dysregulated upon pro-inflammatory activation, including *CDH2* (coding for N-cadherin)^56,57^. We did not, however, observe any significant changes in neural crest *cdh2* mRNA upon LPS + ATP exposure (Supplementary Fig. 5a). Taken together, our results demonstrate that pro-inflammatory activation hampers neural crest migration through downregulation of *CDH1*/E-cadherin, the absence of which is associated with CLP.

### A double hit model affecting neural crest migration

**Fig. 4:**
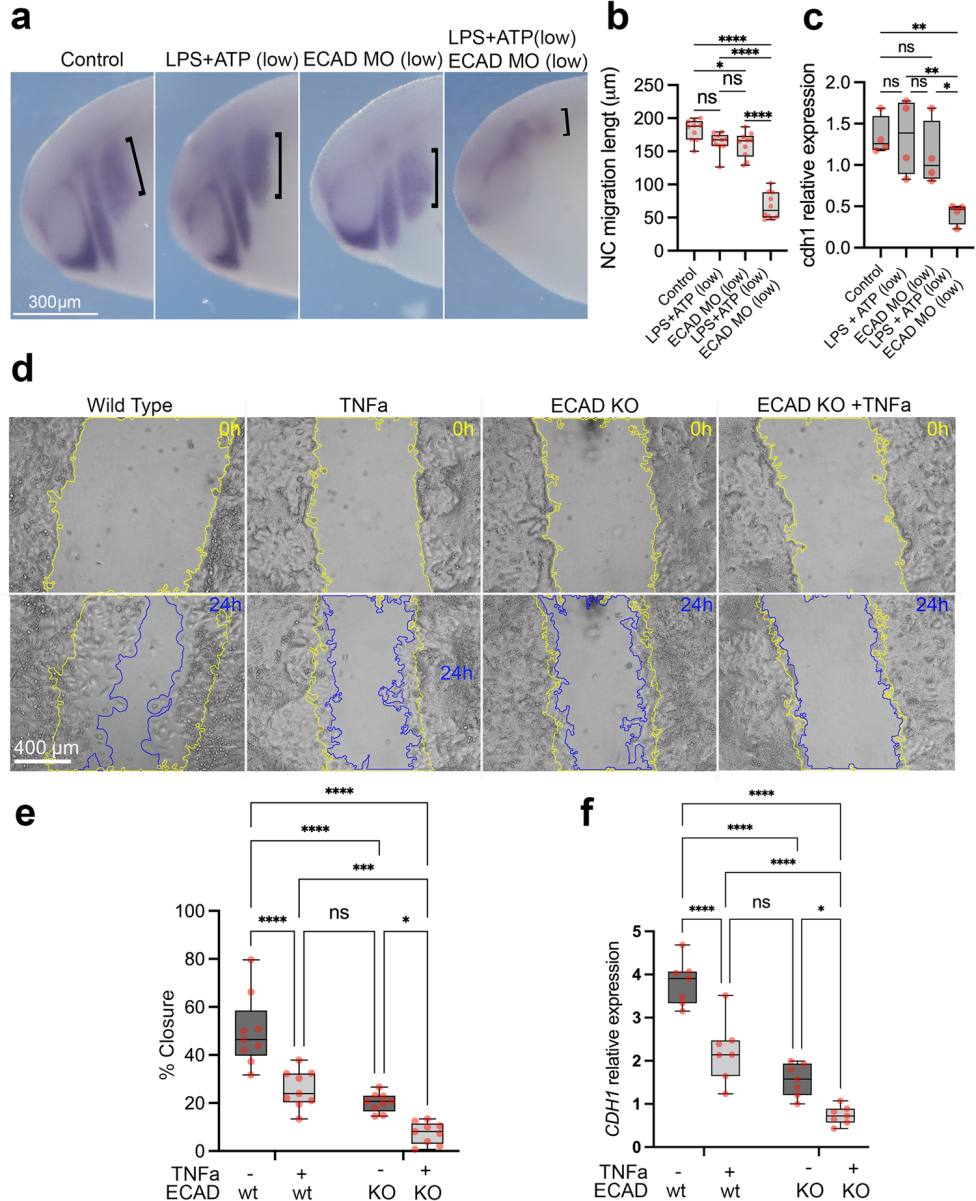
Combined effect of E-cadherin loss-of-function and pro-inflammatory activation supports a 2-hit model for E-cadherin loss in the neural crest. **a** *twist* ISH in control, LPS + ATP low (250ug/ml LPS + 0.1 mM ATP), E-cadherin morpholino low (ECAD MO low; 2 ng ECAD MO) and LPS + ATP low + ECAD MO low treated samples. Neural crest migration length is evidenced by black brackets. **b** Quantification of neural crest migration lengths in control (*n* = 10), LPS + ATP low (*n* = 10), ECAD MO low (*n* = 10) and LPS + ATP low + ECAD MO low (*n* = 10), showing significant reduction of neural crest migration length in the combined LPS + ATP low + ECAD MO low conditions (*p* = 0.002, Two-way ANOVA). **c** cdh1 mRNA levels showing significant reduction in LPS + ATP low + ECAD MO low conditions when compared to controls (*n* = 4 per group; *p* = 0.008. Two-way ANOVA). **d** Scratch assays using hiNCCs in wild type, TNFa exposure, heterozygous E-cadherin knockout (ECAD KO) and combined ECAD KO + TNFa conditions evidencing scratch areas at 0 h (yellow line) and 24 h (blue line) after scratch in each condition. **e** Quantification of scratch percentage of closure in wild type, TNFa exposure, ECAD KO and ECAD KO + TNFa’in hiNCCS after 24 h (*n* = 9 per group). TNFa significantly reduces scratch closure in comparison to wild type (*p* < 0.0001) and combining ECAD KO + TNFa significantly reduces scratch closure in comparison to ECAD KO (*p* = 0.0305) and to TNFa (*p* = 0.0008). Two-way ANOVA. **f** qPCR quantification of CDH1 expression in wild type, TNFa, ECAD KO and ECAD KO + TNFa conditions of hiNCCs (*n* = 7 per group). TNFa significantly reduces *CDH1* expression in comparison to wild type (*p* < 0.0001) and combining ECAD KO + TNFa significantly reduces *CDH1 expression* in comparison to ECAD KO (*p* = 0.0236) and to TNFa (*p* < 0.0001). Two-way ANOVA. Boxplots centre is the median, with bounds representing the 25th and 75th percentile, with and whiskers as minima to maxima. Source data are provided as a Source Data file. *p < 0.05, **p < 0.01, ***p < 0.001 and ****p < 0.0001.

Our hypothesis predicts a 2-hit interaction between a *CDH1* loss-of-function allele plus pro-inflammatory activation leading to a further reduction in *CDH1*/E-cadherin levels, together causing impaired neural crest migration. To test this hypothesis, we applied three different approaches, analysis of *Xenopus* neural crest in vivo, human hiNCCs in vitro and analysis of mouse neural crest in vivo. Firstly, treatment of *Xenopus* embryos with reduced concentrations of LPS + ATP or knockdown of E-cadherin via injection with a morpholino did not affect neural crest migration (Fig. 4a, LPS + ATP low, ECAD MO low), whereas a combination of the same levels of ECAD MO and LPS + ATP significantly impaired neural crest migration in vivo (Fig. 4a–c). Secondly, using the in vitro hiNCCs with either TNFa exposure, *CDH1* heterozygous knockout (ECAD KO/21del ECAD) or a combination of both (ECAD KO + TNFa), showed that the combination of ECAD KO + TNFa resulted in reduced cell migration (wound-healing closure) and lower *CDH1* mRNA levels (Fig. 4d–f). To confirm the requirement of *CDH1*/E-cadherin on neural crest migration we performed transwell assays of wild type and three different *CDH1* heterozygous knockouts (21del ECAD, 7del ECAD, 11del ECAD) hiNCCs, showing decreased migration when cells are exposed to TNFa, and even further reduction in mutant compared to wild type cells (Supplementary Fig. 6). Concordantly, E-cadherin protein levels are significantly reduced in *CDH1* heterozygous knockouts hiNCCs in comparison to control, with lower levels when those cells are exposed to TNFa (Supplementary Fig. 7). No effect in the specification of the cells as neural crest was observed under our treatments as all hiNCCs exhibited >95% SOX10 and SOX9 positive nuclei (Supplementary Fig. 8). In addition, no significant change in caspase 3/7 activity was observed in those cells with or without TNFa exposure, in comparison to control (Supplementary Fig. 9), indicating that our treatments are not inducing apoptosis. To further characterise the effects of exposing hiNCCs to TNFa, the expression of several genes was analysed by qPCR. Pro-inflammatory cytokines *TNFa* and *IL6* are upregulated upon TNFa treatment during the differentiation of hiPSC to hiNCC, and *CDH1* is reduced; neural border (*PAX3* and *TFAP2a*), EMT (*ZEB2*, *TWIST1* and *SNAIL2*) and neural crest genes (*SOX9* and *ETS1*) are mostly unperturbed (Supplementary Fig. 10), confirming that the pro-inflammatory treatment is not affecting specification or differentiation of neural crest cells.

**Fig. 5:**
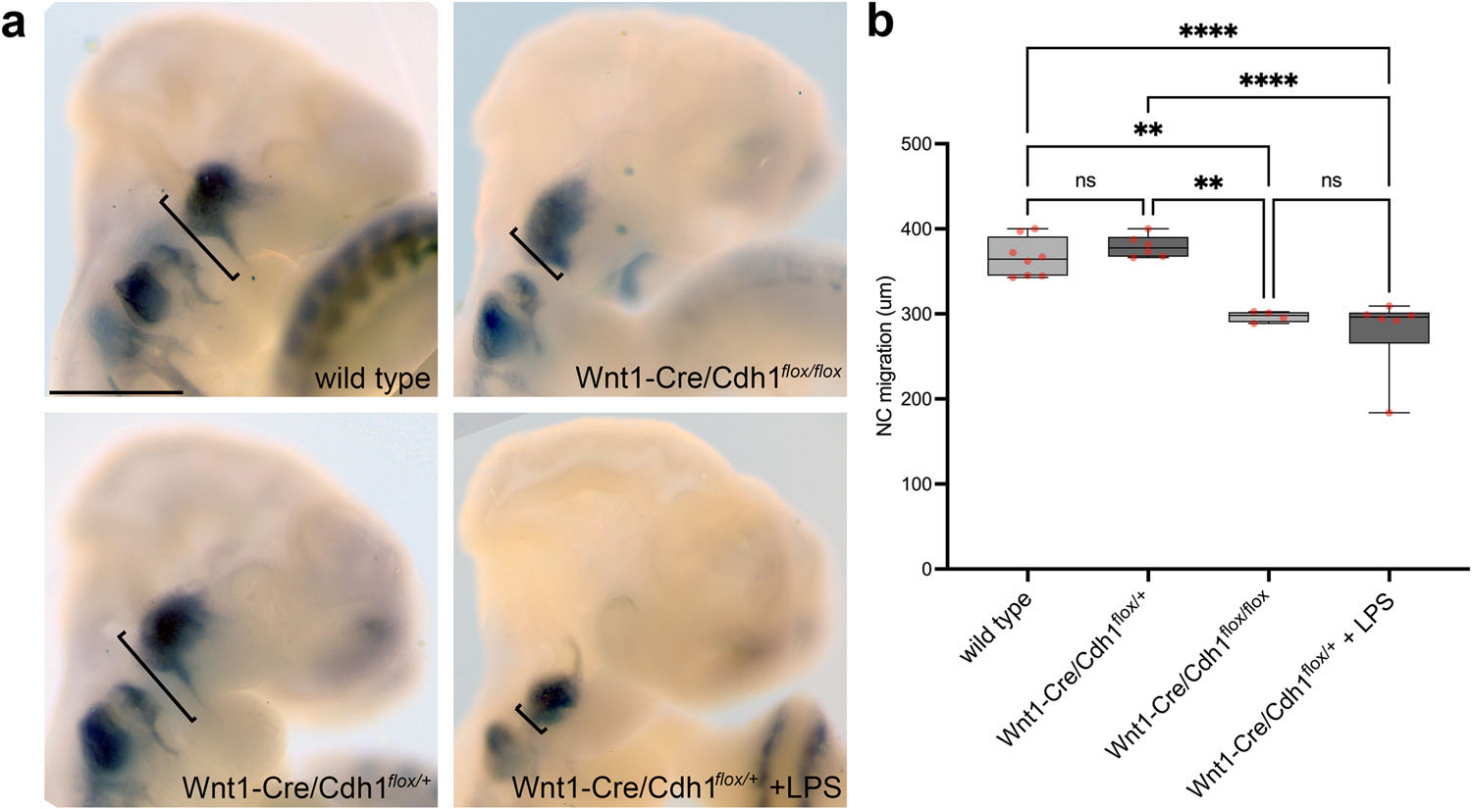
*Cdh1* deficiency in combination with pro-inflammatory activation via maternal immune activation impair neural crest migration in the mouse embryo. **a** In vivo neural crest migration observed in E10.5 mouse embryos by *Sox10* in situ hybridisation. Cranial neural crest migrates ventrally forming streams towards branchial arches in wild type (*n* = 8) and Wnt1-Cre2/Cdh1^flox/+^ (*n* = 6), failing to form such migration streams when Cdh1 is knocked-out in the neural crest (Wnt1-Cre2/Cdh1^flox/flox^, *n* = 4). Upon a pro-inflammatory hit (maternal LPS), Wnt1-Cre2/Cdh1^flox/+^ neural crest fails to migrate towards branchial arches (Wnt1-Cre2/Cdh1^flox/+^ + LPS, *n* = 6). Scale bar = 500um. **b** Quantification of in vivo neural crest migration in mouse embryos showing significant reduction of migration in Wnt1-Cre2/Cdh1^flox/flox^ (*p* = 0.032) and Wnt1-Cre2/Cdh1^flox/+^ + LPS (*p* < 0.0001) embryos in comparison to wild types and Wnt1-Cre2/Cdh1^flox/+^ (*p* = 0.001 and *p* < 0.0001, respectively). Two-way ANOVA. Boxplots centre is the median, with bounds representing the 25th and 75th percentile, with and whiskers as minima to maxima. Source data are provided as a Source Data file. *p < 0.05, **p < 0.01, ***p < 0.001 and ****p < 0.0001.

Lastly, we explored the 2-hit model using *Wnt1-Cre2/Cdh1*^flox/+^ mouse and pro-inflammatory activation through maternal LPS injections. If true, our hypothesis predicts that embryonic lack of E-cadherin together with maternal pro-inflammatory activation would lead to impaired neural crest migration. To gain insight in this model, following the mating of *Wnt1-Cre2* females and *Cdh1*^*f*lox/flox^ males, pregnant females were injected with LPS at 8 and 9 days post-coitum inducing cytokine overexpression in pregnant females and embryos collected at E10.5 for neural crest analysis. First, homozygous embryos lacking E-cadherin (*Wnt1-Cre2 Cdh1*^flox/flox^) exhibited reduced neural crest migration compared with wild type embryos (Fig. 5a, top panels); whereas heterozygous E-cadherin embryos exhibited normal neural crest migration (Fig. 5a, Wnt*1-Cre2 Cdh*1^flox/+^). However, when these heterozygous embryos were exposed to maternal immune activation a reduced neural crest migration was observed (Fig. 5, *Wnt1-Cre2 Cdh1*^flox/+^ + LPS), supporting the 2-hit model.

Taken together, our results support the 2-hit model in which perturbation of *CDH1*/E-cadherin during development can be achieved by both genetic and environmental insults in a complementary fashion. Although not fully explored here, it is possible that pro-inflammatory factors could modulate phenotype penetrance in *CDH1*-linked CLP families. In addition, our results support the notion that a precise level of E-cadherin is required for neural crest migration, consistent with observations on other developmental processes and in cancer cells^58–62^.

### E-cadherin hypermethylation is induced by pro-inflammation

**Fig. 6:**
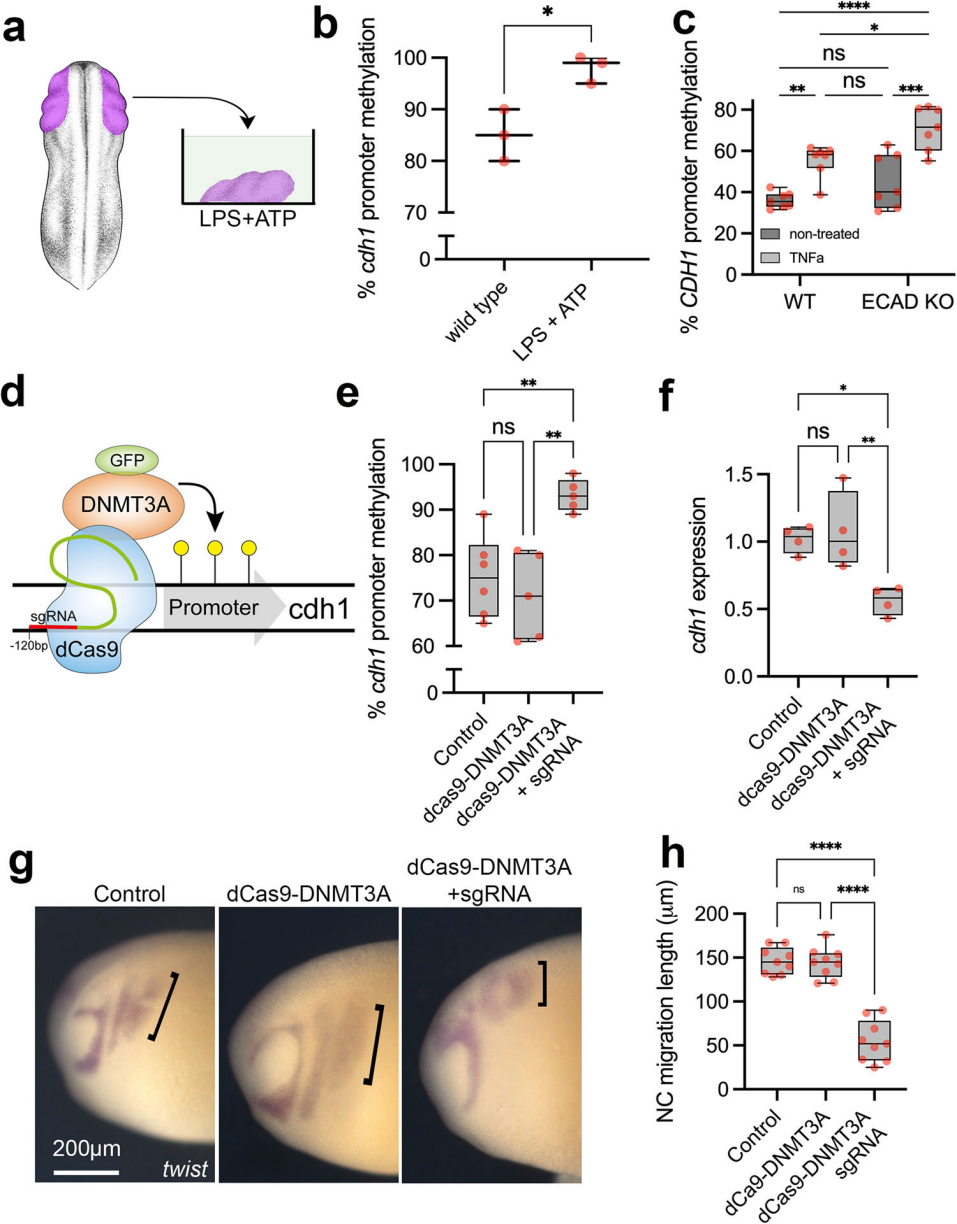
*CDH1* promoter hypermethylation is induced by pro-inflammatory activation and is sufficient to impair neural crest migration. **a** Illustration of neural crest pro-inflammatory activation using neural crest explants and LPS + ATP treatment. *Xenopus* illustrations © Natalya Zahn (2022). **b** Average percentage of *cdh1* promoter methylation in neural crest explants from wild type and LPS + ATP conditions (*N* = 3 explants pools per group, *n* = 10 explants per point). *cdh1* promoter methylation is significantly increased by ~15% in LPS + ATP treated samples in comparison to controls (*p* = 0.0164, two-sided Welch’s *t* test). Data are presented as mean values and minimum to maxima. **c** Average percentage of *CDH1* promoter methylation in hiNCCs under wild type (WT), TNFa, ECAD KO or ECAD KO + TNFa conditions, showing significant *CDH1* promoter methylation in TNFa exposed hiNCCs (*n* = 7 per group; *p* = 0.0048) and ECAD KO + LPS + ATP in comparison to ECAD KO (*p* = 0.0002). Two-way ANOVA. **d** Illustration of dCas9-DNMT3A-GFP system. **e** Average percentage of *cdh1* promoter methylation in neural crest explants from control (non-injected, *N* = 6), dCas9-DNMT3A-GFP (*N* = 5) and dCas9-DNMT3A-GFP + sgRNA (*N* = 5) injected embryos. *cdh1* promoter methylation is increased in dCas9-DNMT3A-GFP + sgRNA injected neural crest in comparison to controls (*p* = 0.0035, Two-way ANOVA). **f** *cdh1* levels in the neural crest from control, dCas9-DNMT3A-GFP and dCas9-DNMT3A-GFP + sgRNA injected embryos, with reduction in dCas9-DNMT3A-GFP + sgRNA in comparison to controls (*p* = 0.009). *N* = 4 per group, pool of neural crest explants *n* = 10 per point. Two-way ANOVA. **g** *twist* ISH in *Xenopus* embryos displaying neural crest migration in control, dCas9-DNMT3A-GFP and dCas9-DNMT3A-GFP + sgRNA injected embryos. Neural crest migration is evidenced by black brackets. **h** Neural crest migration quantification in control, dCas9-DNMT3A-GFP and dCas9-DNMT3A-GFP + sgRNA injected embryos, showing reduction in dCas9-DNMT3A-GFP + sgRNA in comparison to controls (*p* = 0.002). *n* = 9 per group, Two-way ANOVA. Boxplots centre is the median, with bounds representing the 25th and 75th percentile, with and whiskers as minima to maxima. Source data are provided as a Source Data file. *p < 0.05, **p < 0.01, ***p < 0.001 and ****p < 0.0001.

The modulation of genome function in response to environmental changes is frequently established via epigenetic modifiers^63^. In this regard, pro-inflammatory activation is known to modulate gene function via epigenetic changes, including DNA methylation^64, 65^. Furthermore, we have previously demonstrated that penetrant individuals in *CDH1*-linked CLP families display higher *CDH1* promoter DNA methylation levels in comparison to non-penetrant and family control individuals and reanalysed this previous data (Supplementary Fig. 11)^66^. Taking that into account, along with *CDH1* promoter hypermethylation under infectious/pro-inflammatory conditions previously reported^43^, we set out to investigate if pro-inflammatory activation in the neural crest could induce such epigenetic changes. Using neural crest explants exposed to LPS + ATP, we observed a significant increase in *Xenopus cdh1* promoter methylation in comparison to untreated samples, in which the pro-inflammatory condition induced almost full methylation of the *cdh1* promoter (Fig. 6a, b, Supplementary Fig. 12a). Such increased DNA methylation upon pro-inflammatory activation is accompanied with DNA methyltransferase dnmt3a upregulation in the neural crest (Supplementary Fig. 13). Accordingly, hiNCCs exposed to TNFa also displayed increased *CDH1* promoter methylation, both in wild-type and ECAD KO cells (Fig. 6c, Supplementary Fig. 12b). To test the effect of this methylation on E-cadherin expression and neural crest migration we induced *cdh1* hypermethylation targeting the neural crest using dCas9-DNMT3A-GFP mRNA and *cdh1* promoter sgRNA injections in vivo (Fig. 6d, Supplementary Fig. 14). dCas9-DNMT3A-GFP plus sgRNA injected neural crest presented higher *cdh1* promoter methylation levels, as well as reduced *cdh1* mRNA, in comparison to controls and dCas9-DNMT3A-GFP without sgRNA (Fig. 6e, f). Using the same experimental design, we evaluated in vivo neural crest migration under *cdh1* promoter targeted hypermethylation and we observed significant decrease in neural crest migration under dCas9-DNMT3A-GFP plus sgRNA injections (Fig. 6g, h). We therefore concluded that *cdh1* promoter hypermethylation is sufficient to induce neural crest migratory deficiency.

### E-cadherin demethylation rescues pro-inflammatory migration

**Fig. 7:**
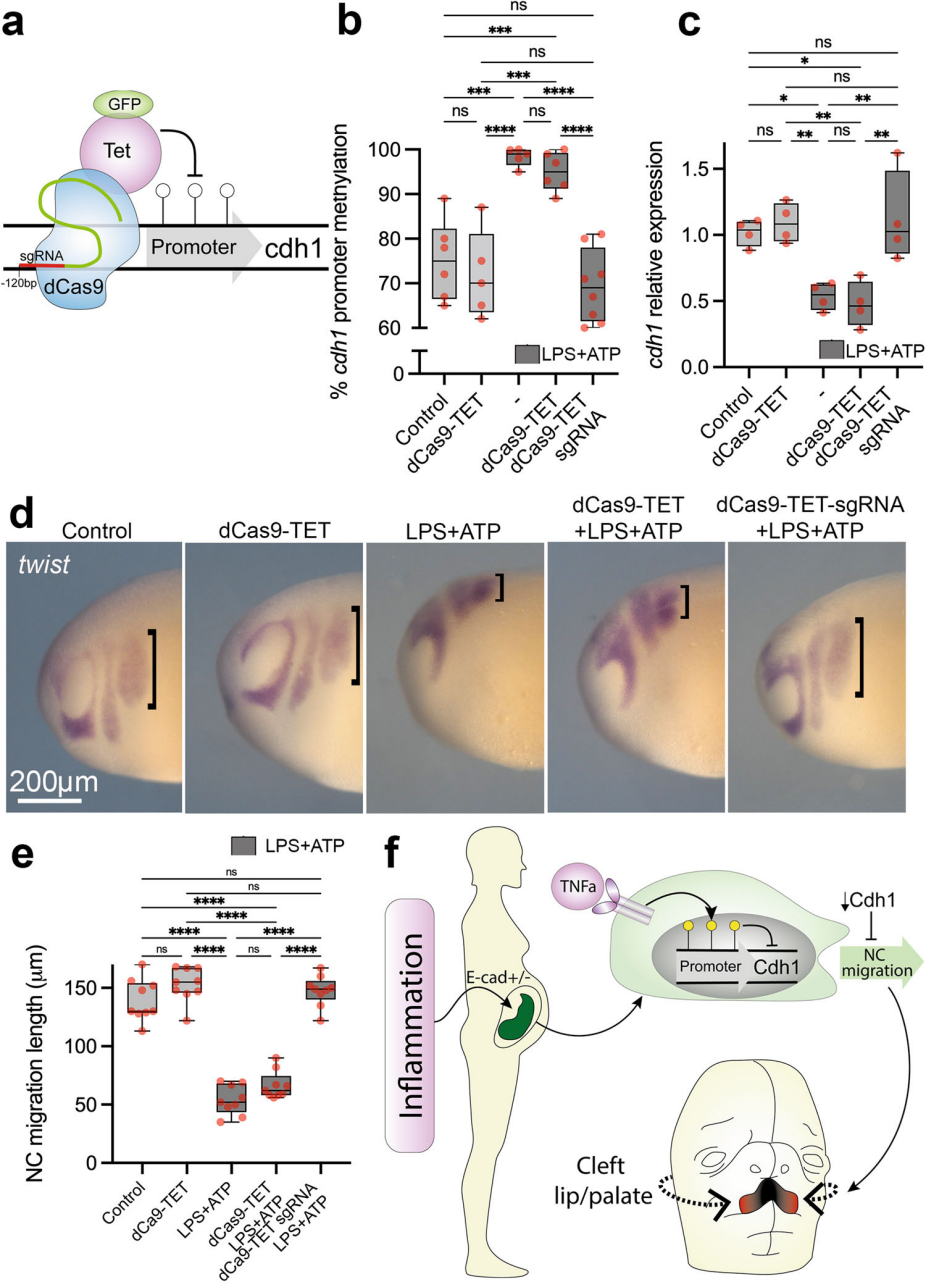
Controlling *cdh1* promoter methylation levels rescues the migratory behaviour of neural crest under pro-inflammatory activation. **a** Illustration of dCas9-TET-GFP system for *cdh1* targeted demethylation in *Xenopus* embryos. **b** Percentage of *cdh1* promoter methylation in neural crest explants from control (*N* = 6), dCas9-TET-GFP (*N* = 5), LPS + ATP (*N* = 5), dCas9-TET-GFP + LPS + ATP (*N* = 6) and dCas9-TET-GFP + sgRNA + LPS + ATP (*N* = 8). While *cdh1* promoter is significantly increased in LPS + ATP and dCas9-TET-GFP + LPS + ATP (*p* = 0.0002 and *p* = 0.008, respectively, Two-way ANOVA), dCas9-TET-GFP + sgRNA injections are efficient in restoring cdh1 promoter methylation under LPS + ATP exposure to control levels (*p* = 0.6010, Two-way ANOVA). **c** PCR quantification of *cdh1* expression in the neural crest from control, dCas9-TET-GFP, LPS + ATP, dCas9-TET-GFP + LPS + ATP and dCas9-TET-GFP + sgRNA + LPS + ATP conditions. *cdh1* expression levels are significantly reduced in LPS + ATP and dCas9-TET-GFP + LPS + ATP (*p* = 0.003 and *p* = 0.008, respectively), and restored to control levels under dCas9-TET-GFP + sgRNA + LPS + ATP (*p* = 0.9356). *N* = 4 per group, pool of neural crest explants *n* = 10 per point. **d** *twist* whole-mount ISH in *Xenopus* embryos (stage 24) displaying neural crest migration in control, dCas9-TET-GFP, LPS + ATP, dCas9-TET-GFP + LPS + ATP and dCas9-TET-GFP + sgRNA + LPS + ATP conditions. Neural crest migration length is evidenced by black brackets. **e** Neural crest migration length quantification in control, dCas9-TET-GFP, LPS + ATP, dCas9-TET-GFP + LPS + ATP. Neural crest migration is significantly reduced in LPS + ATP and dCas9-TET-GFP + LPS + ATP in comparison to controls (*p* > 0.0001 and *p* > 0.0001, respectively) while dCas9-TET-GFP + sgRNA + LPS + ATP restored migration to control lengths (*p* = 0.8904). *n* = 9 per group, Two-way ANOVA. **f** Illustration of pro-inflammatory model affecting neural crest migration. Pro-inflammatory activation during pregnancy would stimulate cytokine production such as TNFa, which in turn would promote *cdh1* promoter hypermethylation and impair neural crest migration, impairing craniofacial development. Boxplots centre is the median, with bounds representing the 25th and 75th percentile, with and whiskers as minima to maxima. Source data are provided as a Source Data file. *p < 0.05, **p < 0.01, ***p < 0.001 and ****p < 0.0001.

If pro-inflammatory activation modulates neural crest *cdh1* levels via DNA methylation, inhibiting *cdh1* promoter methylation during LPS + ATP exposure would rescue the neural crest migratory phenotype. To test this, we used a dCas9-TET1-GFP construct for targeted *cdh1* demethylation with the same sgRNA previously used (Fig. 7a, Supplementary Fig. 14). Under LPS + ATP pro-inflammatory activation, dCas9-TET1_GFP plus sgRNA injections are efficient in reducing *cdh1* methylation levels in the neural crest, as well as upregulating *cdh1* transcript levels (Fig. 7b, c). Most importantly, dCas9-TET1-GFP plus sgRNA injections were able to rescue in vivo neural crest migration under pro-inflammatory conditions (Fig. 7d, e). Hence, both *cdh1*-targeted hypermethylation and hypomethylation assays led us to conclude that *cdh1* epigenetic control via DNA methylation is likely the major consequence of pro-inflammatory activation in the neural crest (Fig. 7f).

## Discussion

We demonstrated a 2-hit model for *CDH1*-linked CLP in which gene loss-of-function, in combination with an environmental hit, namely pro-inflammatory activation, leads to *CDH1* hypomorphism in the neural crest, affecting its migratory properties and resulting in craniofacial malformations.

Pro-inflammatory activation during mouse pregnancy, or maternal immune activation, has been reported to affect neuronal development in mouse embryos, which has been linked to a model of higher susceptibility to the autistic spectrum disorder in humans^67–70^. Despite several contributions in this field, the study of the effects of pro-inflammatory activation in craniofacial development is very limited and this work links pro-inflammatory activation during development affecting the neural crest. We have hypothesised *CDH1*/E-cadherin as a central node affected by pro-inflammatory activation and our results corroborate this idea. We believe, however, that pro-inflammatory activation might affect other genes and pathways that may also contribute to the phenotypes we observed, and such ideas should be investigated.

Importantly, our study corroborates the need of E-cadherin during proper neural crest migration, both in *Xenopus* as in mice embryos, as well as in human iNCCs. We do not address, however, which mechanism the lack of E-cadherin affects neural crest migration by. It has been reported that E-cadherin lack in migratory cells could compromise cell cortex tension, cell polarity or focal adhesion stability^50, 59,71^ and such mechanisms could be involved in the phenotypes here reported. Noteworthily, the experiments conducted here using *Xenopus* embryos aim to target the neural crest by microinjections in specific blastomeres which give rise mainly, but not exclusively, to the neural crest. This raises the question on whether the observed in vivo migratory phenotypes were due to neural crest specific effects or to affected surrounding tissues. To shed light into this question, we have also observed similar reduction in neural crest dispersion using neural crest explants, which demonstrates that neural crest can directly respond to pro-inflammatory factors, reenforcing the idea that such phenotypes are neural crest specific.

Apart from the cellular and developmental phenotypes observed, by using both *Xenopus* and mouse in vivo models for E-cadherin loss-of-function in the neural crest we were able to reproduce craniofacial phenotypes comparable to those in humans. While lip and palatal structures arise from distinct embryonic structures in the frog than in mammals, our results suggest that the lack of E-cadherin affecting neural crest migration can generate malformed craniofacial cartilages in *Xenopus* larvae. Similarly, mouse oral clefting here described are comparable to those previously reported in models for the Cadherin-catenin complex mutations^18^.

Gene–environment interactions are frequently manifested via epigenetic variation^63,72,73^, and here we demonstrated that *CDH1* promoter hypermethylation acts as a mechanism through which pro-inflammatory activation affects gene expression and ultimately cell behaviour during development. Interestingly, we have observed increased DNA methyltransferase dnmt3a expression in *Xenopus* neural crest under LPS-ATP treatment, suggesting pro-inflammatory activation can modulated such genes as reported elsewhere^74,75^ and could be part of the mechanism by which *CDH1* promoter is hypermethylated. Although we acknowledge the limitation of not evaluating other loci than *CDH1* promoter for DNA methylation upon pro-inflammatory activation, our data again suggest a central role for *CDH1* methylation, as revealed by our targeted DNA methylation and demethylation experiments *Xenopus* neural crest, in which *cdh1* methylation control was sufficient to either produce or rescue migratory phenotypes.

Despite widely considered a multifactorial malformation, gene–environment interactions reported in CLP are rarely demonstrated. This is the case of *Msx1* mutation in mice, which has been reported to interact with hypoxia and phenytoin during development and increase penetrance of CLP in the offspring^76^. In this sense, our work explores this multifactorial component of CLP by the investigation of E-cadherin hypomorphism and pro-inflammatory activation during development.

CLP is one of the most common human malformations, which opens the questioning on whether causal factors, either genetic and/or environmental, should be also common in our population. From this perspective, pro-inflammatory conditions during pregnancy could be a common environmental factor, in which maternal infections, diabetes and obesity are potential common causes. We here validate the effects of inflammation on craniofacial development by neural crest migration impairment, opening a route for further investigation of the impacts of pro-inflammatory conditions during development.

Not less important, this study approaches gene–environment interactions by exploring CLP incomplete penetrance. Incomplete penetrance, a frequent phenomenon in autosomal dominant traits, is usually attributed to undetected genetic variants or environmental modulation^22,77^. Here we demonstrate that pro-inflammatory activation could be an environmental factor modulating CLP penetrance. Such findings therefore shed light on how multifactorial conditions can arise from the combination of genetic and environmental factors as well as how incomplete penetrance could be explained for *CDH1*-linked CLP.

## Methods

### Ethics

The use of human data and samples in this study was approved by the Human Research Ethics Committee from the Biosciences Institute (University of Sao Paulo, Brazil) under the protocol 363.876. Biological samples and data regarding from all human research participants were obtained under informed and signed consents from those individuals or legal guardians. The study design and conduct complied with all relevant regulations regarding the use of human study participants and was conducted in accordance with the criteria set by the Declaration of Helsinki.

Mice experiments and procedures were approved by the Animal Research Ethics Committee from the Biosciences Institute (University of Sao Paulo, Brazil) under the protocol 353/2019.

Animal licenses were approved by the Animal Welfare and Ethical Review Board (WERB) at University College London and the UK Home Office. All animal procedures were performed under the ethics standards established by the UK Home Office.

### CDH1 sequencing analysis

Targeted sequencing was performed for families F10626 (individuals II-2, III-1 and III-2), F1842 (III-2) and F8250 (III-2 and IV-1; Supplementary Fig. 1). Libraries were prepared with Ampliseq™ custom panel (Illumina), according to the manufacturer’s instructions, and sequenced using the Illumina MiSeq platform, with MiSeq® Reagent Kit v2 (300 cycle). A mean exon coverage of 503x was achieved (±218 standard deviation), with 98.9% of bases presenting coverage above 20x. Exome and Sanger sequencing performed for family F3788 (Fig. 1a) are described elsewhere^17^. Human *CDH1* isoform NM_004360.3 was adopted as reference for variant nomenclature. Penetrance in those families was estimated using a model previously described^78^.

### Neural crest Cdh1 conditional knockout mice

Mice were kept in cages with water and food *ad lib*, in rooms with controlled temperature and illumination (12 h/12 h light dark cycle), until the moment of euthanasia. Neural crest specific *Cdh1* knockout mice was achieved by the use of *Wnt1-Cre2* mice (129S4.Cg-E2f1Tg(Wnt1-cre)2Sor/J), which expresses Cre-recombinase in neural crest cells (Strain #:022137, The Jackson Laboratory, USA) and LoxP flanked *Cdh1* mice (B6.129-Cdh1tm2Kem/J; Strain #:005319, The Jackson Laboratory, USA). Both mice strains were crossed to obtain *Wnt1-Cre2/Cdh1*^*f*l/+^. Female and male mice were *Wnt1-Cre2/Cdh1*^fl/+^crossed to obtain Wnt1-Cre2/Cdh1^fl/fl^ embryos. Observation of copulatory plugs were made and counted as embryonic day 0.5 (E 0.5). For clefting phenotype, once pregnant mice reached E 15.5, females were euthanized, and embryos collected in PBS and fixed in formalin for further analyses. For pro-inflammatory activation via maternal immune activation in pregnant females, at days 8 and 9 post-coitum females were injected with 100 μg/Kg LPS and females were euthanized at day 10, and embryos collected in PBS and fixed in formalin for further analyses. Adult mice and embryos were genotyped by PCR following The Jackson Laboratories recommendations. As controls, wild-type JB6 or *Wnt1-Cre2* E15.5 embryos were used.

### Xenopus laevis embryo collection

*Xenopus laevis* embryos were obtained as previously described^79^. In brief, ovulation of mature 2–5-year-old females (obtained from NASCO, USA) was induced by injecting 100 IU pregnant mare serum gonadotropin (Intervet) subcutaneously into the dorsal lymph sac. Then, 72 h later, a second injection of 200-300 IU human chorionic gonadotropin (Chorulon, Intervet) was performed. Eggs were fertilised in vitro by mixing with a sperm solution. Testes were provided by the European Xenopus Resource Centre. Embryos were staged according to Nieuwkoop and Faber^80^. Fertilised eggs were dejellied in a solution of 1 g l-cysteine (Sigma-Aldrich) and 500 μl 5 N NaOH in 50 ml H_2_O and maintained in 0.1× MMR or 3/8 normal amphibian media (NAM) at 14.5 °C.

### Morpholino, mRNA and sgRNA microinjections

E-cadherin translation blocking morpholino (ECAD MO) was obtained from GeneTools (USA) as previously described^49^. TNFa and TNFR translation blocking morpholinos were also obtained from GeneTools (USA) and sequences are available at Supplementary Table 2. *cdh1* mRNA was obtained using a cloned *Xenopus cdh1* cDNA construct previously published^50^ and human CDH1 construct (Addgene, plasmid #28009) were used, and in vitro transcription of 5′ capped mRNAs was performed using mMessage mMachine T7 transcription kit (ThermoFisher Scientific). Human CDH1 pathogenic variants c.760G>A and c.2351G>A were added to the original human CDH1 construct by using Q5® Site-Directed Mutagenesis Kit (New England Biolabs), following the fabricant recommendations and confirmation by Sanger sequencing. dCas9-DNMT3A-GFP and dCas9-TET-GFP constructs were obtained from Addgene (pdCas9-DNMT3A-EGFP, plasmid #71666 and pPlatTET-gRNA2, plasmid #82559, respectively). In brief, inserts from both plasmids were subcloned into pCS2+ backbones and in vitro transcription was carried out using T7 RiboMAX™ Express Large Scale RNA Production System (Promega). Transcripts were in vitro 5′capped and polyadenylated using Vaccinia Capping System (NEB) and E. coli Poly(A) Polymerase (NEB), respectively. sgRNA for *Xenopus cdh1* promoter was designed using ChopChop (https://chopchop.rc.fas.harvard.edu/) and synthesised using Precision gRNA Synthesis Kit (ThermoFisher Scientific). *cdh1* sgRNA sequence is available at Supplementary Table 2.

Microinjections were performed using 5 nl solution in a calibrated needle at 8-cell stage embryos into 2 dorsal blastomeres on the right side, while embryos were into NAM 3/8. Eight nanograms of either ECAD MO, TNF MO or TNFR MO were used for microinjections. For ECAD MO low conditions, 2 ng of morpholino was used. For rescue experiments, 50 pg of either *cdh1* mRNA or human CDH1 mRNAs were used. For cdh1 promoter targeted methylation and demethylation assays, 300 pg of dCas9-DNMT3A-GFP or dCas9-TET-GFP were injected, with or without 10 pg of *cdh1* sgRNA. *Xenopus* embryos or incubated at 14.4 °C until developmental stages 20, 24, 25 or 52 for further analyses.

### Microtomography (uCT) scanning and 3-dimensional reconstructions

E15.5 mouse embryos or stage 52 *Xenopus* larvae were used for uCT scan and observation of craniofacial structures. In both cases, embryos were fixed in 4% PFA for 2 h at room temperature and serialy dehydrated in ethanol/PBS. Next, embryos were incubated in 100% Lugol solution (Sigma-Aldrich) overnight at 4 °C. After a 5 min wash in distilled water, embryos were placed in 50 ml plastic tubes and scanned on a Nikon CT Scanner, using 45 mV and 7 mA. Reconstructions were made using CT Pro Scan software and analysed using Voxel software. Measurement of structures was performed on FIJI v.1.53k. Images were coloured using Adobe Photoshop.

### Induced neural crest cells (iNCCs), immunostaining and caspase 3/7 activity measures

iNCCs were generated from healthy induced-pluripotent stem cells (iPSC) and characterised as previously described^81^. iPSCs used in this study were NIBSC8, obtained with the National Institute for Biological Standards and Control (UK). Briefly, iPSCs grown in E8 media (ThermoFisher Scientific) were dissociated as single cells using Tryple Select (ThermoFisher Scientific) and plated at 20.000 cells/cm^2^ and incubated on E6 media (ThermoFisher Scientific) for 4 days to generate neural border cells (NBCs). Next, NBCs were dissociated to single cell and plated at 20.000 cells/cm^2^ density in E6 media added with 1 μM CHIR99021 (Sigma-Aldrich), 20 μM SB431542 (Tocris) and 8 ng/ml basic FGF (ThermoFisher Scientific) for 15 days. Rock-inhibitor Y-27632 (Sigma-Aldrich) was always added at 5uM when cells were harvested.

For *CDH1* heterozygous knockouts (21del ECAD, 11del ECAD and 7del ECAD) iNCCs generation, iPSCs were transfected with dual expressing Cas9 and sgRNA vector pSpCas9(BB)−2A-Puro (Addgene plasmid #62988) containing a sgRNA for *CDH1* exon 3 (Supplementary Table 2). Transfected iPSCs were selected with 1 μg/ml Puromycin for 5 days and survivor colonies were manually isolated for expansion. iPSCs were then Sanger sequenced and frameshift deletions were identified in 3 of the colonies and therefore selected for further assays. iNCCs differentiation was performed as described above.

For immunostaining of those cells, cells were fixed on PFA 4% for 15 min at room termperature and blocked with 2% BSA (PBST) for 1 h at room temperature. Antibody incubation was performed overnight at 4 °C in blocking solution and then cells were washed three times with PBST prior to secondary antibody incubatior. Secondary antibody (AlexaFluor 488 or AlexaFluor 594, Thermofisher Scientific) incubation was performed in blocking solution for 1 h at room temperature using 1:1000 dilution. Primary antibodies used for NCCs were goat anti human/mouse E-cadherin (1:50, AF748, R&D Systems), goat anti human Sox9 (1:100, AB5535, Sigma), mouse anti human Sox10 (1:50, PCRP-SOX10-1D8, DSHB), used following supplier recommendations. Cells were imaged using an EVOS m7000 microscope (Thermofisher Scientific). For apoptotic level quantification in the mentioned iNCCs, we have quantified caspase 3/7 activity by pro-luminescence using the Caspase-Glo® 3/7 Assay System (Promega) following the fabricant recommendations.

### In situ hybridisation and immunostaining

Colorimetric whole-mount in situ hybridisation was performed as previously described^82^. Briefly, *snai2* or *twist* (for *Xenopus* embryos) and *Sox10* (for mouse embryos, a gift from Vassilis Pachnis lab) digoxigenin riboprobes was transcribed using the Riboprobe in vitro Transcription System (Promega, P1420). Embryos were fixed in MEMFA, bleached in 6% hydrogen peroxide, and then incubated with probes for snai2 or twist overnight in hybridisation buffer. Embryos were then washed, blocked with 2% blocking reagent, incubated with 1:3000 anti-digoxigenin-AP antibodies (Roche), and then revealed using NBT/BCIP (Roche) with AP buffer. Embryos were imaged using the Nikon SMZ800N attached to the DS-Fi3 camera (Nikon DS-L4 v.1.4.0.4).

Fluorescent in situ hybridisation was performed as previously described81. In brief, embryos were fixed in MEMFA and incubated with snai2 probe overnight in hybridisation buffer. Embryos were washed, bleached in 3% hydrogen peroxide, incubated with 1:1000 anti-digoxigenin-POD antibody. After washing, embryos underwent the fluorescent POD reaction with Cy5-tyramide solution. For subsequent immunostaining, embryos were dehydrated overnight in 15% sucrose and embedded in 15% sucrose/30% fish gelatin solution, frozen on dry ice and cryosectioned into 30 μm slices. Slides were incubated at 37 °C for 1 h and then at room temperature overnight, washed with PBS, blocked in 10% NGS and then incubated in anti-E-cadherin antibody (1:50; DSHB, 5D3) overnight and then with AlexaFluor 488 anti-mouse (1:1000) and DAPI (1:1000) for 1 h at room temperature. Sections were mounted and imaged on the Leica SP8 confocal microscope where Z-stacks were obtained. Z-stack images were visualised with FIJI v.1.53k.

### Neural crest explants and dispersion assays

Neural crest was dissected as previously described^82^. In brief, using a hair knife, the overlying ectoderm was lifted, and the neural crest removed. Dissection was performed in 3/8 NAM. Neural crest explants were plated on fibronectin-coated plastic dishes with DFA media. Time-lapse microscopy was performed using a Nikon microscope and images were analysed using FIJI v.1.53k in combination with Dispersion Tool for triangulation, dispersion area quantification and speed analysis.

### Pro-inflammatory activation in embryos and cells

*Xenopus* embryos at stage 18 and neural crest explants were exposed to 500 μg/ml lipopolysaccharide (LPS, Sigma-Aldrich) and 1 mM ATP (Sigma-Aldrich) overnight on 0.1 MMR or DFA media, respectively, referred as LPS + ATP treatment. LPS + ATP low conditions were composed by 250 μg/ml LPS and 0.1 mM ATP. Human TNFa protein (ThermoFisher Scientific) was also used for pro-inflammatory activation in *Xenopus* embryos or iNCCs. In *Xenopus* embryos at stage 18, 50 ng of TNFa protein or PBS 1X (control) were injected directly under the epidermis on the neural crest region of one side of the embryo. In iNCCs, 50 ng/ml TNFa protein was added in the final media, while controls were added with the equivalent PBS volume.

### Scratch and transwell assays

For scratch assays, 200,000 iNCCs were plated in 24-well plastic plates coated with Matrigel (Corning) and incubated for 24 h. A central vertical scratch was made in each well using a 20 μl tip and cells were washed with PBS twice and full iNCC media was added. Images were taken immediately after replenishing media (time = 0 h) using a EVOS m7000 microscope (ThermoFisher Scientific) and after 24 h. Images were analysed using FIJI v.1.53k and the automated MRI Wound Healing Tool. Transwell assays were performed using 24-well Thincerts 8um (GBO), where 50.000 cells were plated on matrigel coated Thincerts and left to migrate to the other side for 48 h. Next the cells were fixed on 4% PFA, membranes carefully removed from Thincerts and cells stained with DAPI. After PBS washes, the membranes were imaged on a SP8 confocal and nuclei counted from both sides of membranes using FIJI ImageJ.

### RNA extractions and quantitative PCR (qPCR) analysis of gene expression

RNA extractions were performed using Monarch Total RNA Miniprep Kit (NEB) following the fabricant recommendations. For whole embryo RNA, embryos were individualised in 1.5 ml tubes and lysed using the kit’s Lysis Buffer. For neural crest RNA, pools of 10 neural crest explants were combined in a 1.5 ml tube and lysed as above reported. For iPSCs to NCC differentiation, cell pellets containing 100.000 cells were used, while for iNCC used in the scratch assays the whole well was recovered and lysed in the Lysis buffer. cDNAs were synthesised using SuperScript™ VILO™ Master Mix (ThermoFisher Scientific) using 1 μg of RNA per sample and cDNAs were diluted 1:10 in nuclease-free water. qPCRs reactions were set up in triplicates using 2 μl of cDNA, 10 μl of PowerUp™ SYBR™ Green Master Mix (ThermoFisher Scientific), 100 nM forward and reverse primers and nuclease free water in a 20 μl reaction. Primers for targets used in this study were designed in exon-exon junctions using Primer Blast tool (NCBI) and are described in Supplementary Table 2. Reactions were performed in a QuantStudio 3 qPCR System (ThermoFisher Scientific) using standard parameters. Relative expression values (Delta Ct) were calculated as previously reported^83^, using *ef1a* for *Xenopus* samples and *TBP* for human samples as endogenous controls.

### DNA methylation analysis

Bisulfite sequencing was used for assessing *Xenopus cdh1* and human *CDH1* promoter methylation levels as previously described^84^. In brief, DNA was extracted from neural crest explant pools or iNCC pellets using Monarch® Genomic DNA Purification Kit (NEB) and 500 ng of DNA per sample undergone bisulfite conversion using EpiJET Bisulfite Conversion Kit (ThermoFisher Scientific). Bisulfite-specific PCR was conducted for human *CDH1* promoter and *Xenopus cdh1* promoter, in which primers were designed using MethPrimer^85^ and adaptors were added at the 5’ portion of each primer for peak normalisation83 (Supplementary Table 2). Sanger sequencing of amplicons was performed and peak values for cytosines in CpG positions were called using Chromas Lite Version 2.1.1. Cytosine signals were normalised by a normalisation factor (NF), based on the ratio of the signals for the C and T encoded by the tails of primers. Then, the peak height of each C (Ci) included in the target sequence was corrected for this NF as follow: Cnorm = Ci/NF. Finally, the normalised C signals were used to determine the methylation percentage as in 100 ∗ Cnorm/(Cnorm + T).

### Statistical analysis

Normality in the spread of data for each experiment was tested using the Kolmogorov–Smirnov in Prism 9 (GraphPad). Significances for datasets displaying normal distributions were calculated in Prism 9 with an unpaired two-tailed Student’s *t* tests or One-way or Two-way analysis of variance with post-hoc Tukey’s test for multiple comparisons. Significances for non-normal distributed data were calculated in Prism 9 using two-tailed Mann–Whitney U-tests or Kruskal–Wallis tests with host hoc Dunn’s test for multiple comparisons.

All asterisks of statistical significance or lack or statistical significance (NS) refer to a comparison with the control, unless otherwise indicated.

The authors were not blinded to embryos or cells. The criteria for selection were survival and correct delivery of the injected treatments. Embryos and cells were allocated into experimental groups randomly. No predetermination of sample sizes was performed.

### Illustrations

*Xenopus laevis* illustrations were either adapted from Xenbase (www.xenbase.org RRID:SCR_003280)^86^ using Adobe Illustrator or directly created on Adobe Illustrator.

### Reporting summary

Further information on research design is available in the Nature Portfolio Reporting Summary linked to this article.

## Supplementary information

Supplementary Information

Reporting Summary

## Data Availability

All data produced in this manuscript can be found throughout figures, Supplementary figures and Supplementary tables and in the Source Data file. Source data are provided with this paper. Chop chop sgRNA design: https://chopchop.rc.fas.harvard.edu/. Human *CDH1* accession code: RefSeq NM_004360. Xenopus laevis *cdh1* accession code: RefSeq NM_001172232.1. Xenbase: www.xenbase.org.

## Source data

Source Data
