# Supplementary material for: Neural crest E-cadherin loss drives cleft lip/palate by epigenetic modulation via pro-inflammatory gene–environment interaction
Source: Nat Commun. 2023 May 24;14:2868. doi: 10.1038/s41467-023-38526-1 (PMC10209087; doi:10.1038/s41467-023-38526-1)
Supplement: Supplementary file 1 — Supplementary Information [file 41467_2023_38526_MOESM1_ESM.pdf]

Supplementary Information For:

Neural crest E-cadherin loss drives cleft lip/palate by epigenetic modulation via pro-inflammatory gene-environment interaction.

Lucas Alvizi[1]*, Diogo Nani[2], Luciano Abreu Brito[2], Gerson Shigeru Kobayashi[2], Maria Rita Passos-Bueno[2]* and Roberto Mayor[1,3]*

[1] Department of Cell and Developmental Biology, University College London, Gower Street, London WC1E 6BT, UK

[2] Centro de Estudos do Genoma Humano e Celulas-Tronco, Departamento de Genetica e Biologia Evolutiva, Instituto de Biosciencias, Universidade de Sao Paulo. Sao Paulo, Brazil.

[3] Center for Integrative Biology, Faculty of Sciences, Universidad Mayor, Santiago, Chile

Content:

- **Supplementary Figures 1-16**
- **Supplementary Tables 1-2**

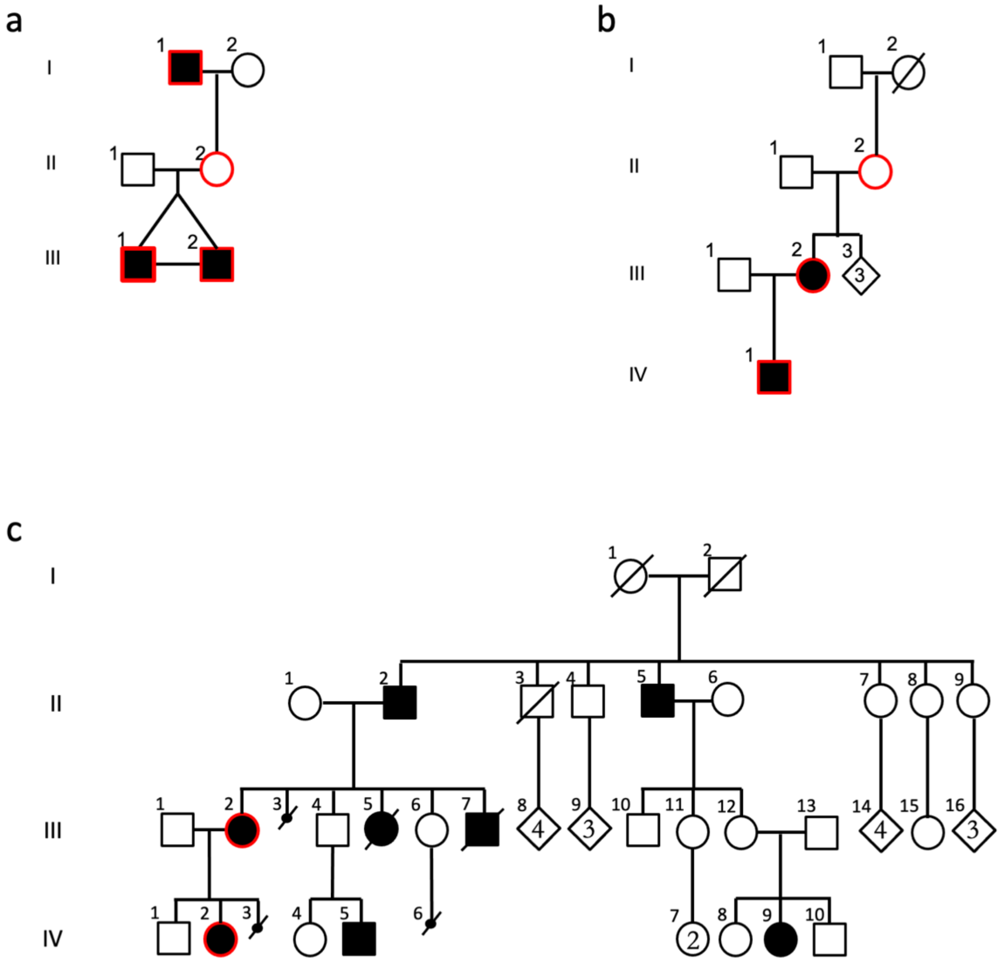

Supplementary Figure 1. ***CDH1* loss-of-function variants are associated with cleft lip/palate with reduced penetrance**. Pedigrees from families displaying CLP and segregating *CDH1* loss-of-function variants. Individuals in red were tested by Sanger sequencing for the following variants: **a**, Family F10626; heterozygous *CDH1* variant c.643delA; p.(Lys215Arhfs*2). **b**, Family F8520; heterozygous *CDH1* variant c.2351G>A, p.(Arg784His). Individual I-2 was reported to have died after complications with gastric cancer. **c**, Family F1842; heterozygous *CDH1* variant c.760G>A, p.(Asp254Asn). Only family F3788 (Figure 1a) was previously reported [17].

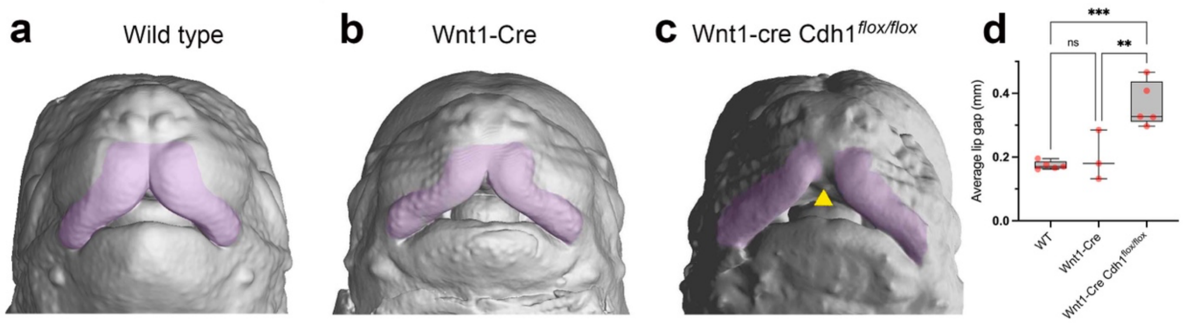

Supplementary Figure 2. **Neural crest *Cdh1* conditional knockout leads to upper lip cleft in mice**. Frontal craniofacial microtomography (μCT) scan reconstructions from E15.5 (**a**) wild-type, (**b**) Wnt1-Cre2 and (**c**) *Wnt1*-Cre2 *Cdh1*$^{flox/flox}$ mice. Yellow arrow indicates wider lip gap in Wnt1-Cre2 *Cdh1*$^{flox/flox}$ E15.5 mouse embryo. **d**, Wnt1-Cre2 *Cdh1*$^{flox/flox}$ E15.5 mouse embryos (n = 50 have significant wider upper lip gaps in comparison to controls (n=5) (p = 0,0009) and to Wnt1-Cre2 (n = 3) (p = 0,0068). One-way ANOVA. Boxplots centre is the median, with bounds representing the 25th and 75th percentile, with and whiskers as minima to maxima. Source data are provided as a Source Data file. *p<0,05, **p<0,01, ***p<0,001 and ****p<0,0001.

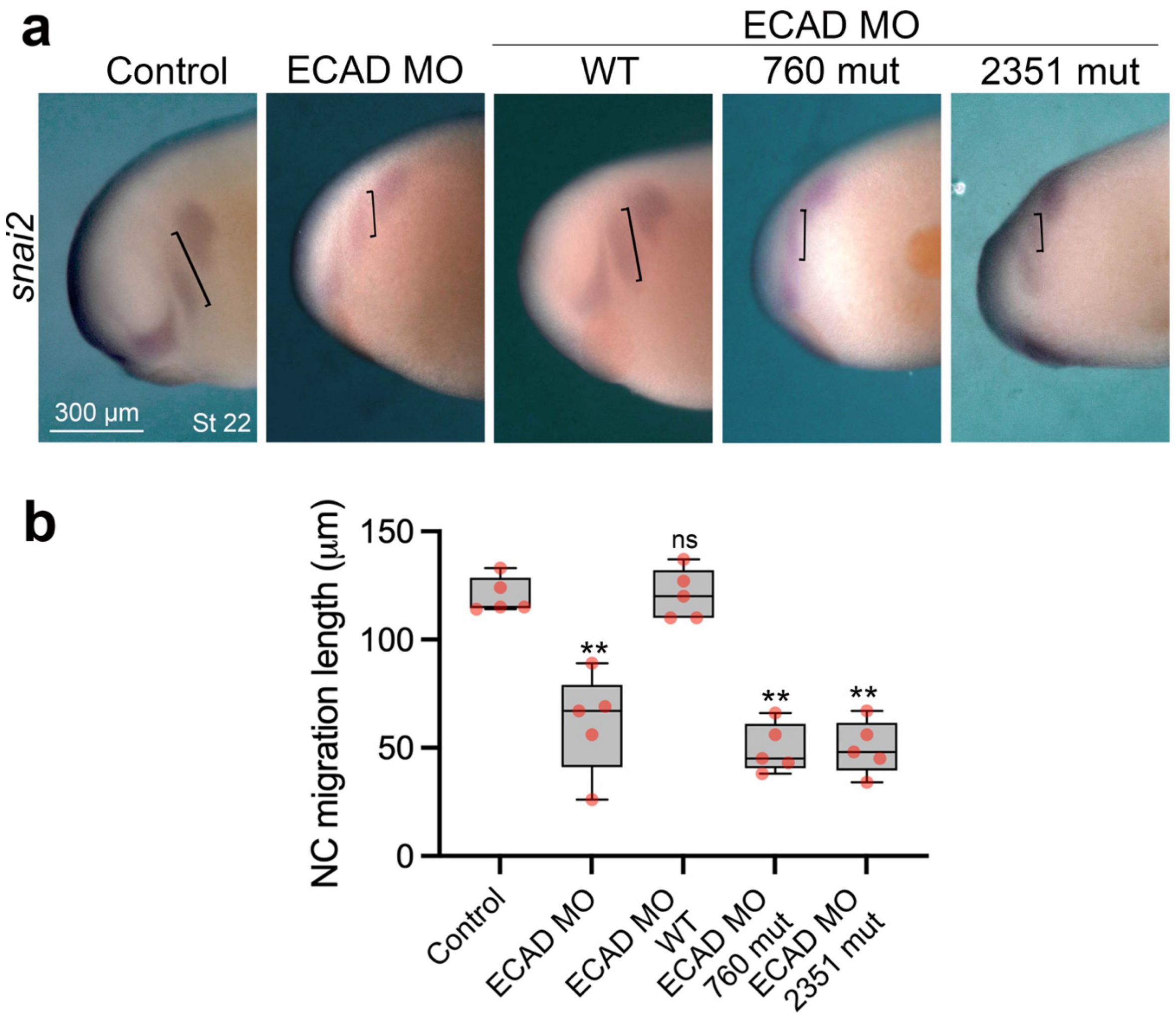

Supplementary Figure 3. CDH1 mRNA bearing human mutations fail to rescue in vivo neural crest migration. a. Neural crest migration revealed by in situ hybridisation of snai2 is reduced when E-cadherin morpholino (ECAD MO) is injected and can be rescued by co-injection of wild-type human E-cadherin mRNA (WT). On the other hand, co-injection of ECAD MO with human E-cadherin mRNAs bearing CLP mutations (760 mut and 2351 mut) fail to rescue normal neural crest migration when compared to controls. **b**. Neural crest migration quantification showing significant reduction of migration length in embryos either injected with ECAD MO ($p<0{,}0001$) or co-injected with ECAD MO and mutant E-cadherin mRNAs 760 mut ($p<0{,}0001$) or 2351 mut ($p<0{,}0001$) ($n = 5$ per group, One-way ANOVA). Boxplots centre is the median, with bounds representing the 25th and 75th percentile, with and whiskers as minima to maxima. Source data are provided as a Source Data file. *p<0,05, **p<0,01, ***p<0,001 and ****p<0,0001.

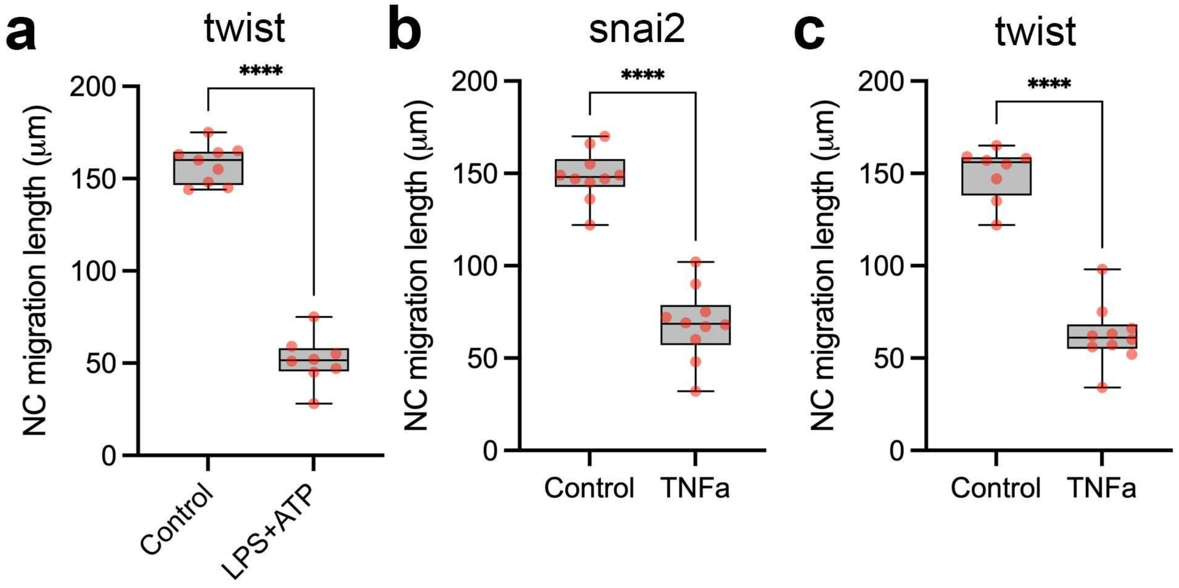

Supplementary Figure 4. **Pro-inflammatory activation leads to *in vivo* neural crest migration deficiency**. **a**, LPS + ATP treated *Xenopus* embryos (n = 8) displayed significant reduction in neural crest migration in comparison to control (n = 9), when quantified using *twist* whole-mount ISH (p < 0,0001, two-sided t-test with Welch's correction). Boxplots centre is the median, with bounds representing the 25th and 75th percentile, with and whiskers as minima to maxima. **b**, Stage 18 embryos injected with human TNFa protein into the neural crest region had significant reduction in neural crest migration in comparison to control, when quantified using *snai2* whole-mount ISH (n = 10 per group; p < 0,0001, two-sided t-test with Welch's correction). Boxplots centre is the median, with bounds representing the 25th and 75th percentile, with and whiskers as minima to maxima. **c**, Stage 18 embryos injected with human TNFa protein (n = 10) into the neural crest region had significant reduction in neural crest migration in comparison to controls (n = 8), when quantified using *twist* whole-mount ISH (p < 0,0001, two-sided t-test with Welch's correction). Boxplots centre is the median, with bounds representing the 25th and 75th percentile, with and whiskers as minima to maxima. Source data are provided as a Source Data file. *p<0,05, **p<0,01, ***p<0,001 and ****p<0,0001.

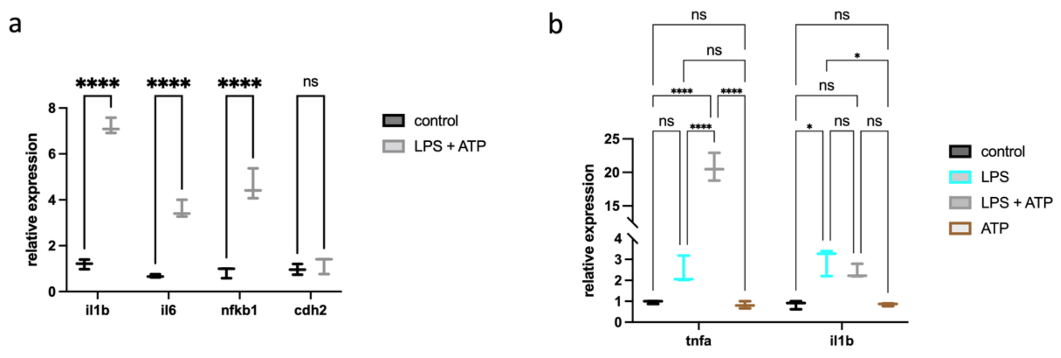

Supplementary Figure 5. **LPS+ ATP treatment in *Xenopus* embryos is efficient in inducing pro-inflammatory cytokine transcription**. **a**, Real-time quantitative PCR in LPS+ATP treatment of stage 15 *Xenopus* embryos induces significant upregulation of *il1b* (p < 0,0001), *il6* (p < 0,0001) and *nfkb1* (p < 0,0001). No significant changes are observed for *cdh2* transcript levels (p = 0,9044). n = 3 per group. One-way ANOVA. Data are presented as mean values and minimum to maxima. **b**, LPS+ATP significantly upregulates *tnfa* transcription in comparison to controls (p < 0,0001), LPS (p < 0,0001) or ATP alone (p < 0,0001). LPS alone does not significantly increase *tnfa* expression in comparison to controls (p = 0,2478), which is similar for ATP (p = 0,9900). *il1b* transcripts were significantly upregulated upon LPS+ATP (p = 0,0378) or LPS (p = 0,0437). No differences were found when comparing *il1b* levels between LPS+ATP and LPS (p = 0,9630) or between ATP to controls (p > 0,9999). n = 3 per group. Two-way ANOVA. Data are presented as mean values and minimum to maxima. Source data are provided as a Source Data file. *p<0,05, **p<0,01, ***p<0,001 and ****p<0,0001.

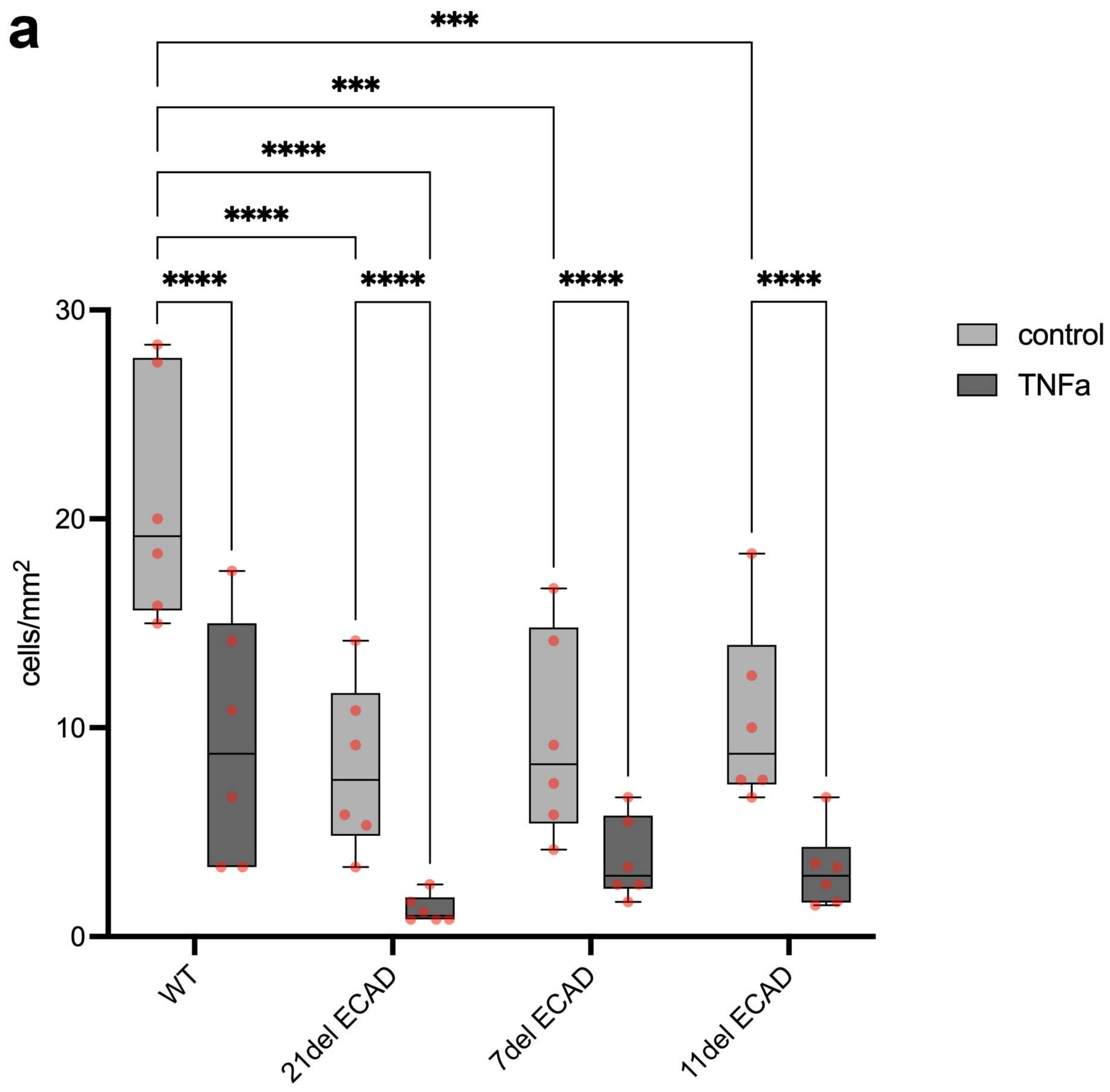

Supplementary Figure 6. Human induced neural crest cells (iNCCs) have reduced cell migration in transwell assays when exposed to pro-inflammation in combination to *CDH1* deletions. iNCCs exposed to the pro-inflammatory cytokine TNFa or bearing heterozygous CDH1 deletions (21del ECAD, 7del ECAD or 11del ECAD) migrate significantly less than controls (WT); Transwell migration is further impaired when deletions are combined with TNFa pro-inflammatory stimuli, with significant reduction in migration when compared to untreated paired groups. n = 6 per group, Two-way ANOVA. Boxplots centre is the median, with bounds representing the 25th and 75th percentile, with and whiskers as minima to maxima. Source data are provided as a Source Data file. *p<0,05, **p<0,01, ***p<0,001 and ****p<0,0001.

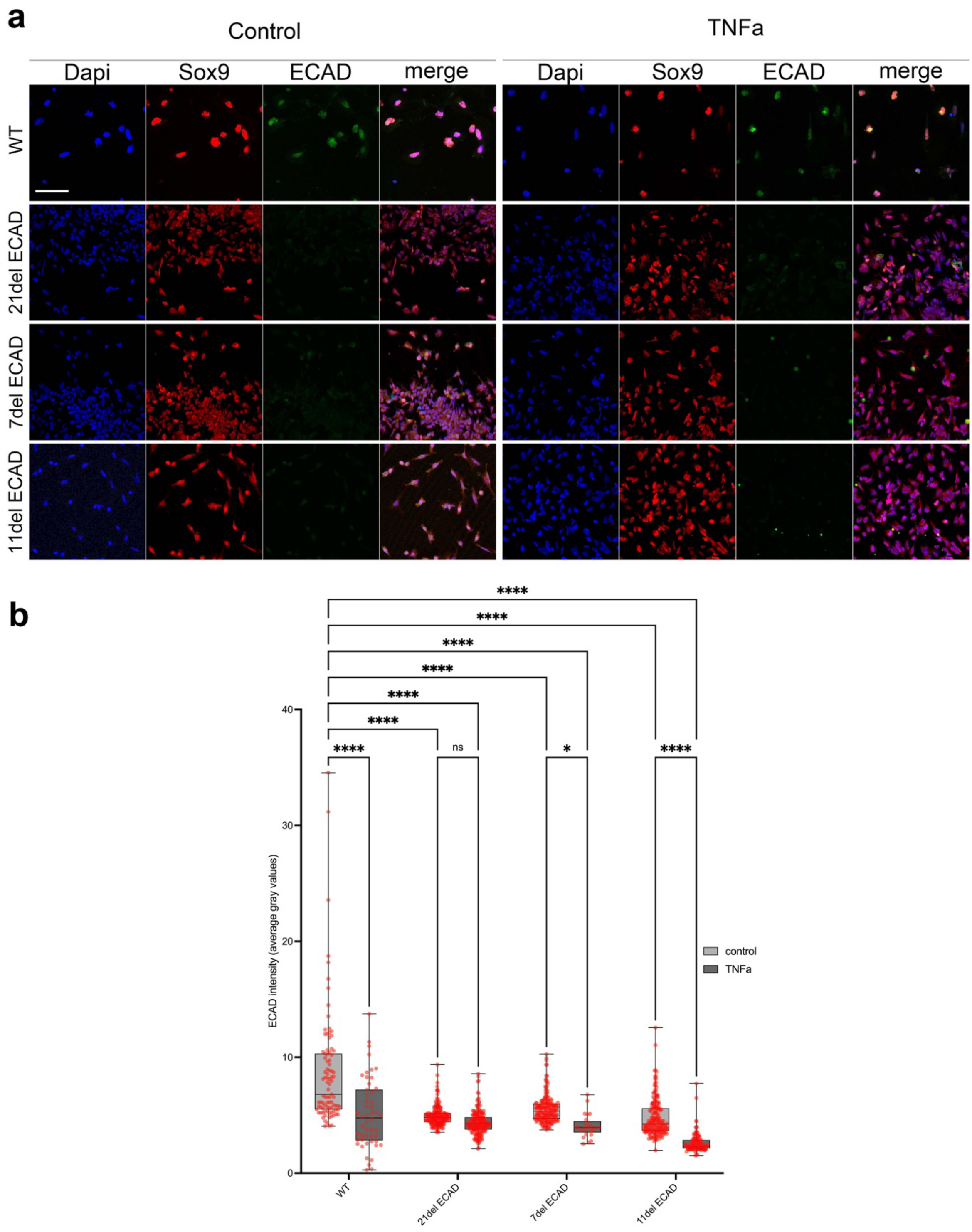

Supplementary Figure 7. Human induced neural crest cells (iNCCs) have reduced E-cadherin protein levels upon pro-inflammatory activation. **a**. Imunnofluorescence stainings for DAPI (blue) Sox9 (red) and ECAD in control cells (WT) and CDH1 heterozygous mutants (21del ECAD, 7del ECAD and 11del ECAD), with pro-inflammatory activation (TNFa) or without (control). Scale bar 80μm. **b**. Quantification of E-cadherin protein levels (average grey values) in immunofluoresce stainings from controls (WT) and CDH1 heterozygous

mutants (21del ECAD, 7del ECAD and 11del ECAD), with pro-inflammatory activation (TNFa) or without (control), showing significant reduction of E-cadherin levels in mutants and pro-inflammatory conditions (TNFa). WT control n = 91 cells; 21del ECAD control n = 197 cells; 7del ECAD control n = 196 cells; 11del ECAD control n = 194 cells; WT + TNFa n = 50 cells (p<0,0001); 21del ECAD + TNFa n = 197 cells (p<0,0001); 7del ECAD + TNFa n = 25 cells (p<0,0001); 11del ECAD + TNFa n = 120 cells (p<0,0001). Two-way ANOVA. Boxplots centre is the median, with bounds representing the 25th and 75th percentile, with and whiskers as minima to maxima. Source data are provided as a Source Data file. *p<0,05, **p<0,01, ***p<0,001 and ****p<0,0001.

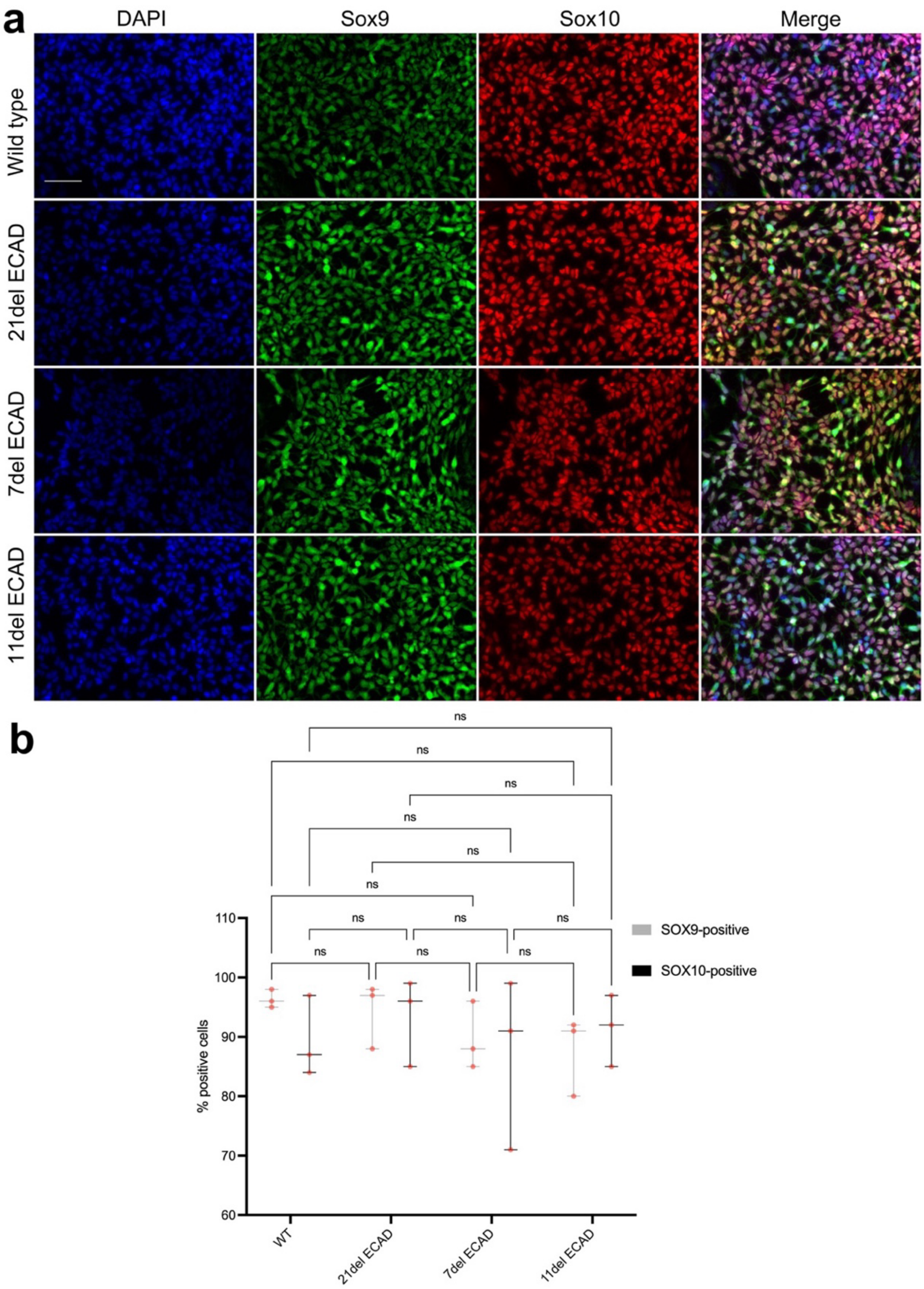

Supplementary Figure 8. Human induced neural crest cells (iNCCs) show similar neural crest markers expression independently of *CDH1* heterozygous deletions. **a**. Immunofluorescence stainings for DAPI (blue), neural crest markers SOX9 (green) and SOX10 (red) in control iNCCs (WT) and iNCCs bearing CDH1 heterozygous deletions (21del ECAD, 7del ECAD and 11del ECAD). Scale bar =100μm. **b**. Quantification of positive cells (positive nuclei) in analysed iNCCs showing no significant difference in average percentage of SOX9 or SOX10 positive cells in comparison to controls (n = 3 per group). Two-way ANOVA. Data are presented as mean values and minimum to maxima. Source data are provided as a Source Data file.

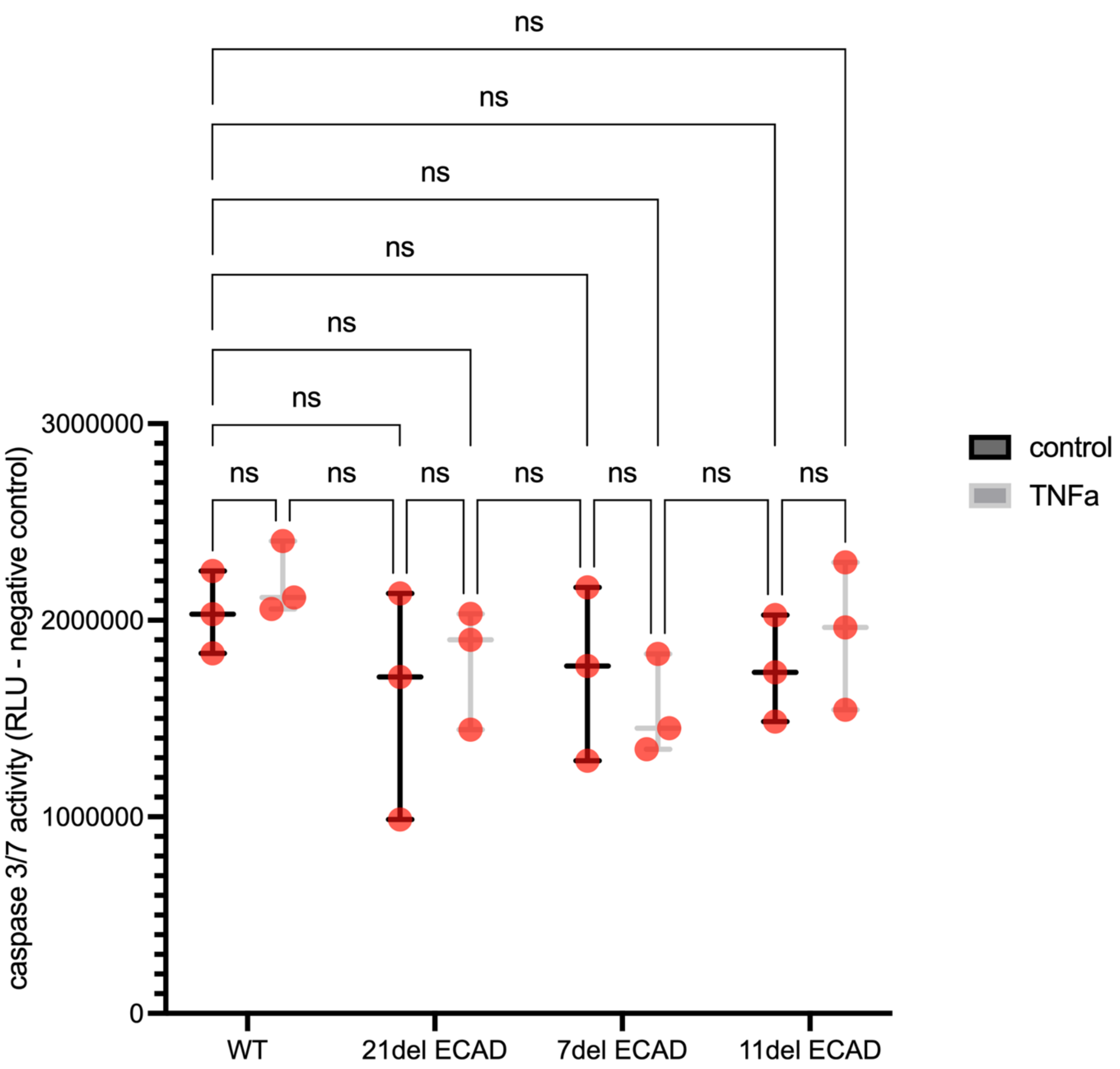

Supplementary Figure 9. Caspase 3/7 activity in human induced neural crest cells (iNCCs). Control (WT) and CDH1 heterozygous iNCCs (21del ECAD, 7del ECAD and 11del ECAD) revealed no significant differences in apoptotic levels as measured by caspase 3/7 activity, independently of pro-inflammatory activation (TNFa). ($n = 3$ per group; $p>0.05$, Two-way ANOVA). Data are presented as mean values and minimum to maxima. Source data are provided as a Source Data file

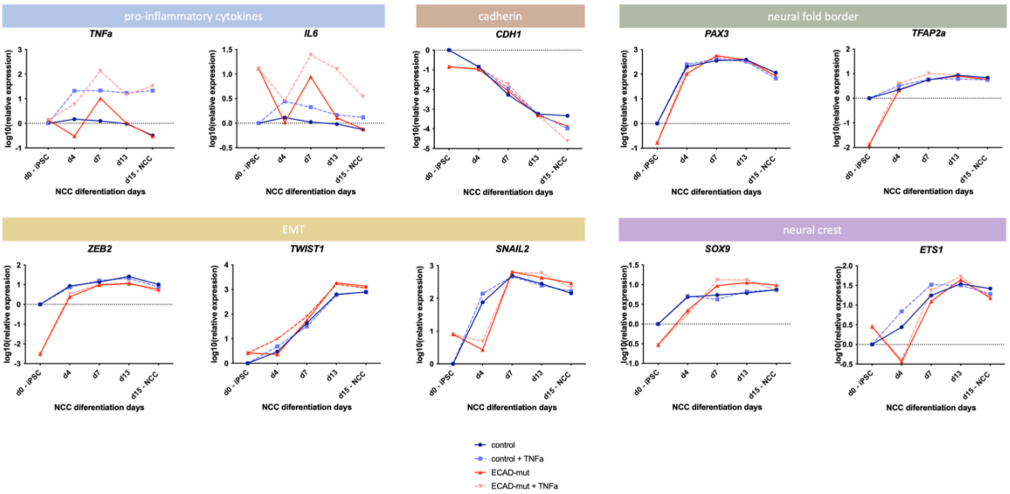

Supplementary Figure 10. **Pro-inflammatory cytokines, *CDH1*, neural border markers, EMT markers and neural crest markers profile during iPSC to iNCC differentiation**. Visual representation of expression levels from various markers (log10 of relative expression) in iPSC, day 4 (d4), day 7 (d7), day 13 (d13) and day 15 (d15-NCC) from control (blue), control + TNFa (dashed light blue), ECAD KO (red) and ECAD KO + TNFa (dashed light red). In display are mRNA expression profiles from pro-inflammatory cytokines (light blue bar, *TNFa* and *IL6*), cadherin (brown bar, *CDH1*), neural fold border markers (green bar, *PAX3* and *TFAP2a*), EMT markers (yellow bar, *ZEB2*, *TWIST1* and *SNAI2*) and neural crest markers (purple bar, *SOX9* and *ETS1*). Under TNFa *protein* exposure, cells upregulate *TNFa* or *IL6* throughout most of the differentiation days. *CDH1* is mostly downregulated in d15-NCC under ECAD KO + TNFa. EMT markers peak similarly in all conditions during d7 or d13. Neural crest markers are similarly expressed at the final stage of differentiation (d15-NCC) in all conditions. Source data are provided as a Source Data file.

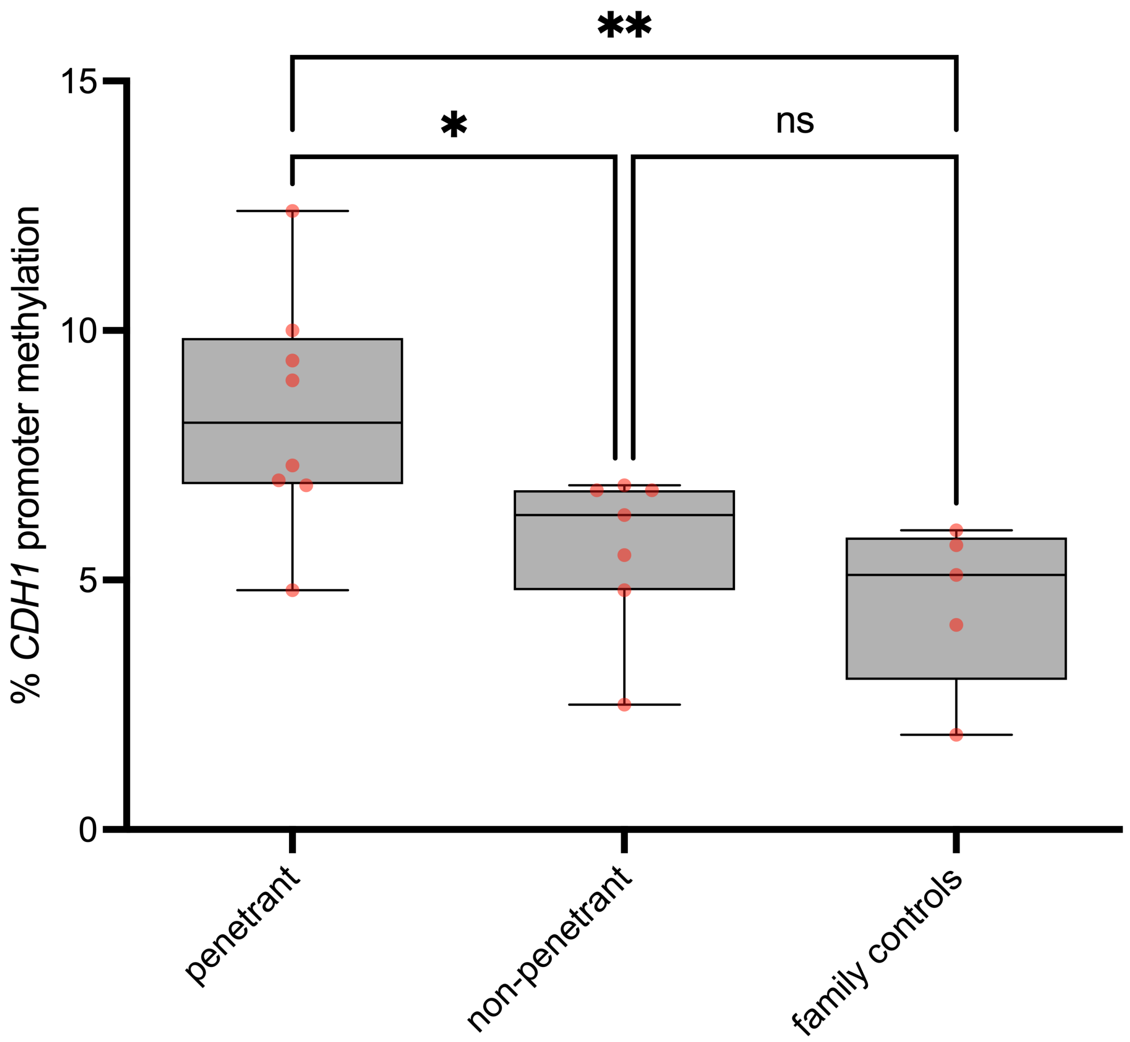

Supplementary Figure 11. *CDH1* promoter methylation is higher in penetrant CLP individuals than in non-penetrant and controls in CDH1-linked CLP families. CDH1 promoter levels data were reanalysed from Brito et al, 2015 (Figure 1a, Supplementary Figure 1), in which penetrant (CLP individuals with *CDH1* pathogenic variants, n = 8; penetrans vs non-penetrant p = 0,0406; penetrant vs family controls p = 0,0088) and non-penetrant (individuals with *CDH1* pathogenic variants with no CLP, n = 7; non-penetrant vs family controls p = 0,61) and family controls (individuals from those families with neither CLP nor *CDH1* pathogenic variants, n = 5) had whole-blood DNA analysed for *CDH1* promoter methylation. One-way ANOVA. Boxplots centre is the median, with bounds representing the 25th and 75th percentile, with and whiskers as minima to maxima. Source data are provided as a Source Data file. *p<0,05, **p<0,01, ***p<0,001 and ****p<0,0001.

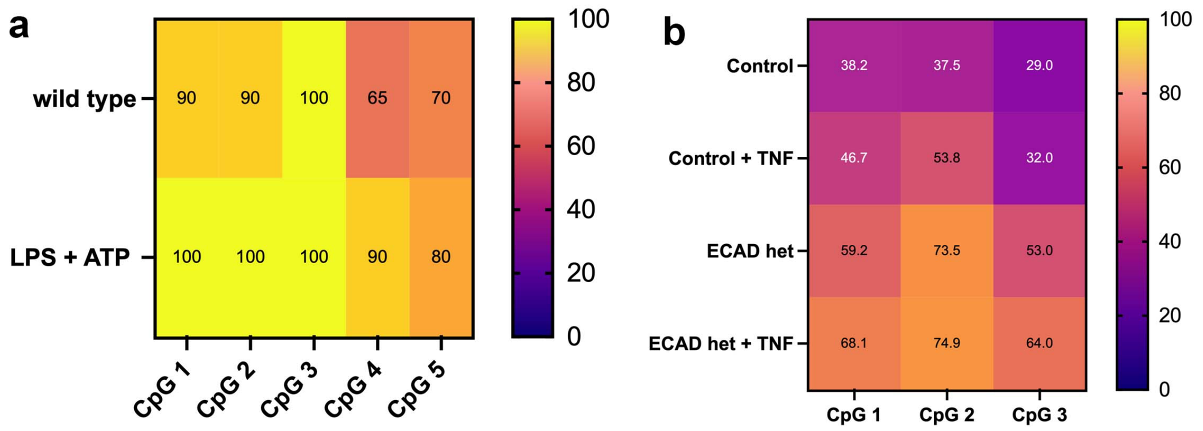

Supplementary Figure 12. Pro-inflammatory activation induces CDH1 promoter hypermethylation in Xenopus neural crest cells and human induced neural crest cells (iNCCs). a. Average CpG methylation in cdh1 promoter in control (wild type) or pro-inflammatory activated (LPS+ATP) Xenopus neural crest, in which 5 CpGs were analysed. All 5 CpGs show increased methylation levels in LPS+ATP condition, with CpG 4 showing a higher increase (25%). **b**. Average CpG methylation in CDH1 promoter in control and *CDH1* heterozygous (ECAD het) under pro-inflammatory activation (TNF), in which data from 3 CpGs were analysed. CpG methylation upon TNFa stimuli is higher in all CpGs when compared to the non-treated pair. Source data are provided as a Source Data file.

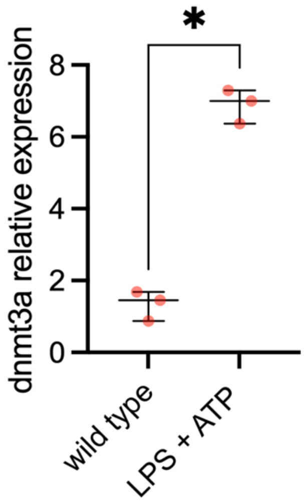

Supplementary Figure 13. Pro-inflammatory activation induces dnmt3a upregulation in Xenopus neural crest. Real time quantitative PCR of DNA methyltransferase dnmt3a in Xenopus neural crest explants shows significant increase in dnmt3a transcription in pro-inflammatory activated explants (LPS+ATP) in comparison to controls (wild type). n = 3 per group. Data are presented as mean values and minimum to maxima. Source data are provided as a Source Data file. *p<0,05, **p<0,01, ***p<0,001 and ****p<0,0001.

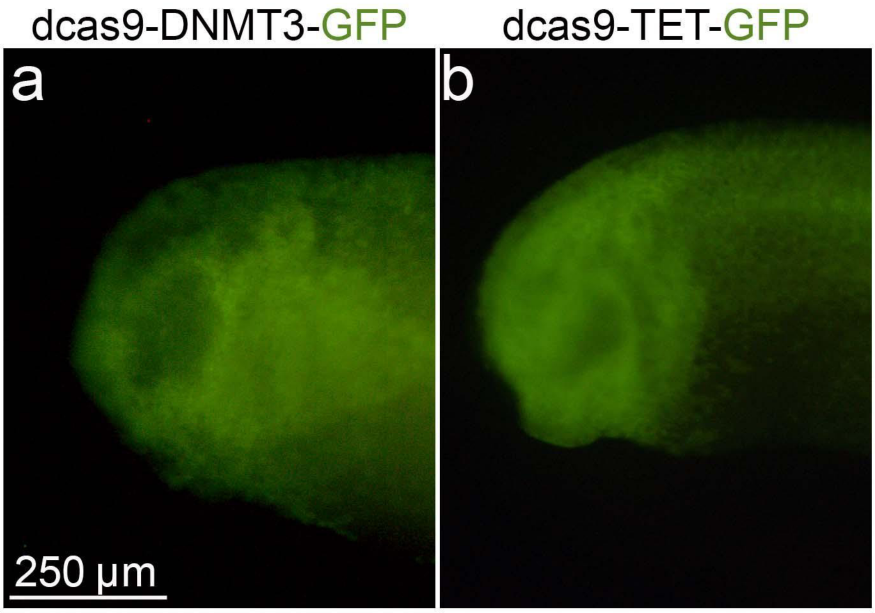

Supplementary Figure 14. **GFP expression in *Xenopus* embryos injected with dCas9-DNMT3A-GFP or dCas9-TET-GFP mRNA**. GFP can be detected in either (**a**) dCas9-DNMT3A-GFP (n = 30) or (**b**) dCas9-TET-GFP (n = 25) mRNA injected embryos. Images taken at stage 25. GFP is mostly observed in the anterior part of the embryo (head), in which neural crest cells are migrating to.

Supplementary Table 1: *CDH1* loss-of-function variants or missense variants found in Cleft lip / palate families. *CDH1* variants were found either by exome or targeted sequencing in four CLP families. CADD (Combined Annotation Dependent Depletion) phred score represented for missense variants, as well as ACMG (The American College of Medical Genetics and Genomics) interpretation of variant, segregation and gnomAD (The Genome Aggregation Database) frequency of listed variant. NA = non-analysed. NR: not reported.

Family	Variant	Type	CADD phred score	ACMG interpretation	Segregation	gnomAD frequency
F10626	c.643delA; p.(K215fs)	del. *frameshift*	NA	Pathogenic	Complete (3 individuals)	NR
F1842/ F3788	c.760G>A; p.(D254N)	*missense*	32	Pathogenic	Complete (2 individuals)	NR
F8520	c.2351G>A; p.(R784H)	*missense*	34	Pathogenic	NA	0.4x10-5

Supplementary Table 2. Oligonucleotides used for gene expression, genotyping and targeted methylation analysis. List of oligonucleotide sequences used for real time quantitative PCR (RT-qPCR), bisulfite sequencing (BS), single guide RNAs (sgRNA) or genotyping.

Organism	Usage	Target	Forward	Reverse
Xenopus laevis	RT-qPCR	*cdh1*	CGACCTTTGGACAGAGAAGC	GCACAGAGCCTTCAAAGACC
		cdh2	CAGCAACGATGGCTTAGTGA	ATTGTAACGGAGACGGTTGC
		ef1a	GACTACCCTCCTCTTGGTCG	CTGAGACCAATCCTTCAGCCC
		tnfa	AACAGGAATTCCACCTGGCG	ATCTTGCTCTCATCCTTTTCAGC
		il1b	TGGGACACCCATTAAAGAGC	TCTCCCATGTCGTCCTTCATTC
		nfkb1	ACGTTGTCTGGGGCTACTAC	GTGCAAATTCCCTCTGTCTCC
		snai2	AAGGGACCGTTACTTGTGCG	TGGGGAGATGATCACTGTATGG
	BS	*cdh1 promorter*	GAGATTYGGGGAGATTTTTTTGT	CATTTAAATACTAAAATACATACCTCAACA
	sgRNA	*cdh1 promorter*	TTTACAGCACTGTAACAGCC	
Human	RT-qPCR	*CDH1*	CCATTCAGTACAACGCCCAACCC	CACAGTCACACACGCTGACCTC
		TBP	GTGACCCAGCATCACTGTTTC	GCAAACCAGAAACCCTTGCG
		PAX3	AAGCCCAAGCAGGTGACAAC	CTCGGATTTCCCAGCTGAAC
		TFAP2a	CTCCGCCATCCCTATTAACAAG	GACCCGGAACTGAACAGAAGA
		TWIST	CAATGACATCTAGGTCTCCGGGCCC	TACGCCTTCTCGGTCTGGAGGATG
		SNAI2	TCTGCGGCAAGGCGTTTTCCAG	GCAAATGCTCTGTTGCAGTGAGGG
		ZEB2	AATGCACAGAGTGTGGCAAGGC	CTGCTGATGTGCGAACTGTAGG
		ETS1	TCAAGGACTATGTGCGGGAC	TTGGTCCACTGCCTGTGTAG
		SOX9	AGCGAACGCACATCAAGAC	CTGTAGGCGATCTGTTGGGG
		IL6	GAGGAGACTTGCCTGGTGAAA	TGGCATTTGTGGTTGGGTCA
		TNFa	TCCCCAGGGACCTCTCTCTA	GAGGGTTTGCTACAACATGGG
	BS	*CDH1*	CAGGAAACAGCTATGACCATTTTTAGTGATGGGAGTGGG	TGACTGGTACGTACCAACACCTTTCTTAACATAACAAATACCT
	sgRNA	*CDH1*	TGAACCCTCAGCCAATCAG	
	Genotyping	*CDH1*	CCCGGTTCCATCTACCTTTCC	TTTCCAACCCCTCCCTACTCC
