# Supplementary material for: Neural crest E-cadherin loss drives cleft lip/palate by epigenetic modulation via pro-inflammatory gene–environment interaction
Source: Nat Commun. 2023 May 24;14:2868. doi: 10.1038/s41467-023-38526-1 (PMC10209087; doi:10.1038/s41467-023-38526-1)
Supplement: Supplementary file 2 — Reporting Summary [file 41467_2023_38526_MOESM2_ESM.pdf]

nature portfolio

Corresponding author(s): Lucas Alvizi
Maria Rita Passos-Bueno
Roberto Mayor

Last updated by author(s): May 3, 2023

Reporting Summary

Nature Portfolio wishes to improve the reproducibility of the work that we publish. This form provides structure for consistency and transparency in reporting. For further information on Nature Portfolio policies, see our Editorial Policies and the Editorial Policy Checklist.

Statistics

For all statistical analyses, confirm that the following items are present in the figure legend, table legend, main text, or Methods section.

n/a	Confirmed	
☐	☒	The exact sample size (*n*) for each experimental group/condition, given as a discrete number and unit of measurement
☒	☐	A statement on whether measurements were taken from distinct samples or whether the same sample was measured repeatedly
☐	☒	The statistical test(s) used AND whether they are one- or two-sided<br>*Only common tests should be described solely by name; describe more complex techniques in the Methods section.*
☒	☐	A description of all covariates tested
☐	☒	A description of any assumptions or corrections, such as tests of normality and adjustment for multiple comparisons
☐	☒	A full description of the statistical parameters including central tendency (e.g. means) or other basic estimates (e.g. regression coefficient) AND variation (e.g. standard deviation) or associated estimates of uncertainty (e.g. confidence intervals)
☐	☒	For null hypothesis testing, the test statistic (e.g. *F*, *t*, *r*) with confidence intervals, effect sizes, degrees of freedom and *P* value noted<br>*Give P values as exact values whenever suitable.*
☒	☐	For Bayesian analysis, information on the choice of priors and Markov chain Monte Carlo settings
☒	☐	For hierarchical and complex designs, identification of the appropriate level for tests and full reporting of outcomes
☒	☐	Estimates of effect sizes (e.g. Cohen's *d*, Pearson's *r*), indicating how they were calculated

Our web collection on statistics for biologists contains articles on many of the points above.

Software and code

Policy information about availability of computer code

Data collection	Data was collected using Microsoft Office Excel (360) and exported to GraphPad Prism 9.
Data analysis	Data was analyzed using GraphPad Prism 9 for statistics and graph design.

For manuscripts utilizing custom algorithms or software that are central to the research but not yet described in published literature, software must be made available to editors and reviewers. We strongly encourage code deposition in a community repository (e.g. GitHub). See the Nature Portfolio guidelines for submitting code & software for further information.

Data

Policy information about availability of data

All manuscripts must include a data availability statement. This statement should provide the following information, where applicable:

- Accession codes, unique identifiers, or web links for publicly available datasets
- A description of any restrictions on data availability
- For clinical datasets or third party data, please ensure that the statement adheres to our policy

All data produced in this manuscript can be found throughout Figures, Supplementary Figures and Supplementary Tables and in the Source Data file. Source data are provided with this paper.
Chop chop sgRNA design: https://chopchop.rc.fas.harvard.edu/)
Human CDH1 accession code: RefSeq NM_004360

Xenopus laevis cdh1 accession code: RefSeq NM_001172232.1

Research involving human participants, their data, or biological material

Policy information about studies with human participants or human data. See also policy information about sex, gender (identity/presentation), and sexual orientation and race, ethnicity and racism.

Reporting on sex and gender	Sex and/or gender-based analysis was not performed.
Reporting on race, ethnicity, or other socially relevant groupings	Human participants were Brazilian individuals from either South-east or north-east Brazil.
Population characteristics	Human research participants were Brazilian individuals with cleft lip/palate, diagnosed by clinicians and clinical geneticists, and their corresponding relatives. As in this study we used segregation analysis of genetic variants, co-variates as age do not play a role. Ages vary from 68 to 2 years in the reported heredograms.
Recruitment	Human research participants were recruited during humanitarian missions of Operation Smile Brazil and families were followed up and ascertained by the authors. No potential biases were identified as those individuals belong to cleft and lip families and no distinct cohorts were compared.
Ethics oversight	Human Research Ethics Committee from the Biosciences Institute (University of Sao Paulo, Brazil) under the protocol 363.876. Informed consents were obtained by either Lucas Alvizi or Luciano Brito (co-authors) during appointments with those individuals. The documentation (approved by the Human Research Ethics Committee from the Biosciences Institute, University of Sao Paulo Brazil) was read and explained to all human research participants and signed by them. This documentation is retained at the Biosciences Institute, University of Sao Paulo Brazil, under the responsibility of Maria Rita Passos-Bueno (co-author).

Note that full information on the approval of the study protocol must also be provided in the manuscript.

Field-specific reporting

Please select the one below that is the best fit for your research. If you are not sure, read the appropriate sections before making your selection.

☒ Life sciences ☐ Behavioural & social sciences ☐ Ecological, evolutionary & environmental sciences

For a reference copy of the document with all sections, see nature.com/documents/nr-reporting-summary-flat.pdf

Life sciences study design

All studies must disclose on these points even when the disclosure is negative.

Sample size	No sample size calculation/estimation was performed. Sample size related to human research participants was limited to the number of families and individuals in each family. Animal embryos (Xenopus or mouse) were obtained with no maximum calculations, however a minimum of 3 was always aimed, as this is the minimal sample size for statistics. Similarly, for human cell lines we established a minimum of 3 cell lines for comparison
Data exclusions	No data exclusion was performed.
Replication	Replication was performed at least in three independent batches and were successful. Another replication strategy adopted in this manuscript followed corroboration using different biological models. For example, results found in Xenopus embryos were replicated in human cell lines and in mouse embryos whenever possible.
Randomization	No randomization was performed as it was not relevant for this study. Samples were evaluated in regards to their phenotype and frequencies or specific measurements were taken and compared to test the relevant hypothesis.
Blinding	Blinding was not performed during group allocation or analysis, as all samples were named after their groups and or phenotypes. We do not believe however blinding was relevant to the study since phenotypes were consistently reproducible amongst different strategies.

Reporting for specific materials, systems and methods

We require information from authors about some types of materials, experimental systems and methods used in many studies. Here, indicate whether each material, system or method listed is relevant to your study. If you are not sure if a list item applies to your research, read the appropriate section before selecting a response.

Materials & experimental systems

n/a	Involved in the study	
☐	☒	Antibodies
☐	☒	Eukaryotic cell lines
☒	☐	Palaeontology and archaeology
☐	☒	Animals and other organisms
☒	☐	Clinical data
☒	☐	Dual use research of concern
☒	☐	Plants

Methods

n/a	Involved in the study	
☒	☐	ChIP-seq
☒	☐	Flow cytometry
☒	☐	MRI-based neuroimaging

Antibodies

Antibodies used

anti- Xenopus E-cadherin (DSHB, 5D3), AlexaFluor 488 anti-mouse (A28175, Thermofisher Scientific), anti-digoxigenin-AP (11093274910, Sigma), anti-digoxigenin-POD (11207733910, Sigma), goat anti human/mouse E-cadherin (AF748, R&D Systems), goat anti human Sox9 (AB5535, Sigma), mouse anti human Sox10 (PCRP-SOX10-1D8, DSHB)

Validation

anti- Xenopus E-cadherin (DSHB, 5D3): Validation using IHC and WB as reported in J Cell Biol
. 1989 Jun;108(6):2449-58. doi: 10.1083/jcb.108.6.2449.
AlexaFluor 488-anti-mouse (A28175, Thermofisher Scientific) - The sensitivity and specificity of each lot is confirmed using ELISA. IF also performed.
anti-digoxigenin-AP (11093274910, Sigma) and anti-digoxigenin-POD (11207733910, Sigma): Dot-blot, ELISA, WB and ISH.
human/mouse E-cadherin (AF748, R&D Systems): Simple Western lane view shows lysates of 4T1 mouse breast cancer cell line, P19 mouse embryonal carcinoma cell line, A431 human epithelial carcinoma cell line, and MCF-7 human breast cancer cell lines.
anti human Sox9 (AB5535, Sigma): This highly published antibody has been validated in IHC & WB.
mouse anti human Sox10 (PCRP-SOX10-1D8, DSHB): HuProt array screen and WB as in Nat Methods
. 2018 May;15(5):330-338. doi: 10.1038/nmeth.4632. Epub 2018 Mar 19.

Eukaryotic cell lines

Policy information about cell lines and Sex and Gender in Research

Cell line source(s)

NIBSC8 human iPSC (purchased at the National Institute for Biological Standards and Control -UK). Sex: Female

Authentication

None of the cell lines were authenticated, as this is a common IPSC line used by diverse studies.

Mycoplasma contamination

All cell lines tested negative for Mycoplasma using the MycoAlert Kit (Lonza).

Commonly misidentified lines
(See ICLAC register)

No commonly misidentified lines were used in this work.

Animals and other research organisms

Policy information about studies involving animals; ARRIVE guidelines recommended for reporting animal research, and Sex and Gender in Research

Laboratory animals

Females Xenopus laevis (from Xenopus One);
Mus musculus:
Wnt1-Cre2: 129S4.Cg-E2f1Tg(Wnt1-cre)2Sor/J. Strain #:022137 (Jax laboratories, USA)
Cdh1 LoxP (Cdh1 flox): B6.129-Cdh1tm2Kem/J. Strain #:005319, Jax laboratories,USA)
Xenopus laevis were 2 to 5 years old and Mus musculus were 3 to 8 months old.

Wild animals

No wild animals were used in this work.

Reporting on sex

No sex-based analysis were performed in this study in regards to laboratory animals. To the best of our knowledge, our data does not apply to sex-based phenotypes. Therefore, no sex-based information has been collected.

Field-collected samples

No field-collected samples were used in this work.

Ethics oversight

Mice experiments and procedures were approved by the Animal Research Ethics Committee from the Biosciences Institute (University of Sao Paulo, Brazil) under the protocol 353/2019. Animal licenses were approved by the Animal Welfare and Ethical Review Board (WERB) at University College London and the UK Home Office. All animal procedures were performed under the ethics standards established by the UK Home Office.

Note that full information on the approval of the study protocol must also be provided in the manuscript.
